# PE homeostasis rebalanced through mitochondria-ER lipid exchange prevents retinal degeneration in *Drosophila*

**DOI:** 10.1371/journal.pgen.1009070

**Published:** 2020-10-16

**Authors:** Haifang Zhao, Tao Wang

**Affiliations:** 1 National Institute of Biological Sciences, Beijing, China; 2 Tsinghua Institute of Multidisciplinary Biomedical Research, Tsinghua University, Beijing, China; New York University, UNITED STATES

## Abstract

The major glycerophospholipid phosphatidylethanolamine (PE) in the nervous system is essential for neural development and function. There are two major PE synthesis pathways, the CDP-ethanolamine pathway in the endoplasmic reticulum (ER) and the phosphatidylserine decarboxylase (PSD) pathway in mitochondria. However, the role played by mitochondrial PE synthesis in maintaining cellular PE homeostasis is unknown. Here, we show that *Drosophila pect* (phosphoethanolamine cytidylyltransferase) mutants lacking the CDP-ethanolamine pathway, exhibited alterations in phospholipid composition, defective phototransduction, and retinal degeneration. Induction of the PSD pathway fully restored levels and composition of cellular PE, thus rescued the retinal degeneration and defective visual responses in *pect* mutants. Disrupting lipid exchange between mitochondria and ER blocked the ability of PSD to rescue *pect* mutant phenotypes. These findings provide direct evidence that the synthesis of PE in mitochondria contributes to cellular PE homeostasis, and suggest the induction of mitochondrial PE synthesis as a promising therapeutic approach for disorders associated with PE deficiency.

## Author summary

Phosphatidylethanolamine (PE) is a critical component of all cellular membranes, and maintaining cellular PE homeostasis is critical for survival and function of cells especially neuronal cells. There are two major PE synthesis pathways in eukaryotes, the CDP-ethanolamine pathway in the endoplasmic reticulum (ER) and the PSD pathway in mitochondria. From a genome-wide genetic screen for genes necessary for photoreceptor cell survival, we identified mutations in the gene *pect*. These mutants displayed defective visual responses, aberrant phospholipid composition, and light-independent retinal degeneration in the absence of mitochondrial defects. Genetic interactions indicated that a deficiency in cellular PE caused retinal degeneration in *pect* mutants. Strikingly, increasing PE synthesis through the PSD pathway restored levels of cellular PE as well as all PE species, and rescued *pect* mutant phenotypes, which is dependent on the exchange of lipids between mitochondria and the ER. Our work provides direct evidence that maintaining cellular PE levels is critical for neuronal function and integrity, suggests that PE synthesized in the mitochondria can be exported to the ER to compensate for deficiencies in cellular PE in neurons, and highlights a fundamental function of mitochondria in cellular phospholipid homeostasis.

## Introduction

Phospholipids are a critical component of all cellular membranes. Among these, phosphatidylethanolamine (PE) is the most abundant phospholipid in the nervous system, regulating neuronal development and function [1, 2]. PE plays important roles in membrane fusion, the regulation of cholesterol homeostasis, mitochondria function, and autophagy [3–6]. Thus, maintaining PE homeostasis is critical for neurons to survive and function [7].

The two pathways involved in PE biosynthesis are the CDP-ethanolamine pathway [8] and the phosphatidylserine decarboxylase (PSD) pathway [9, 10]. The CDP-ethanolamine pathway is the major PE synthesis pathway in eukaryotes. Components of this pathway localize to the endoplasmic reticulum (ER), including ethanolamine kinase, CTP:phosphoethanolamine cytidylyltransferase (*PCYT2*), and 1,2-diacylglycerol ethanolamine phosphotransferase. By contrast, the PSD pathway operates exclusively on the outer leaflet of the mitochondrial inner membrane [10, 11]. Both PE synthesis pathways are essential for cell function and viability, but the PSD pathway is particularly important for mitochondrial morphology and function [5, 6, 12, 13]. PE generated by the PSD pathway in mitochondria is also exported to other cellular organelles, and the non-vesicular lipid transfer of PE from mitochondria to the ER occurs at the endoplasmic reticulum-mitochondria contact sites (ERMCS) [14–19]. However, since the CDP-ethanolamine pathway produces most cellular PE, the physiological importance of mitochondrial PE export is unknown.

Recently, a hypomorphic mutation in *PCYT2* (a component of the CDP-ethanolamine pathway) was shown to cause recessive forms of progressive neurodegeneration, namely spastic paraplegia disorders [7]. Homozygous mutations in PSD genes cause a range of conditions, including skeletal dysplasia, early-onset retinal degeneration, hearing loss, microcephaly, and intellectual disability [20–22]. It is clear therefore that both PE biosynthesis pathways are crucial for the function and survival of neurons.

The *Drosophila* visual system is a powerful genetic model for dissecting the mechanisms of neuronal function and related neurodegenerative diseases [23, 24]. In *Drosophila* photoreceptor neurons, knocking down the PSD pathway results in neurodegeneration, and disrupting the CDP-ethanolamine pathway leads to the loss of synaptic vesicles [25, 26]. In the present study, we conducted a forward genetic screen in *Drosophila* to identify genes required for photoreceptor survival and isolated loss-of-function mutations in the gene *pect*, which encodes *phosphoethanolamine cytidylyltransferase*. Loss of PECT activity led to aberrant phospholipid composition, defective phototransduction, and light-independent retinal degeneration. Importantly, *pect* mutant phenotypes, including cellular PE levels, and photoreceptor cell function and survival, were rescued by induction of the mitochondrial PSD pathway. These studies demonstrate that the PE synthesis pathway is required for neuronal function and integrity. We further provide strong genetic evidence that the PSD pathway contributes to cellular PE levels and works with the CDP-ethanolamine pathway to maintain PE homeostasis.

## Results

### A forward genetic screen reveals that *pect* is required for photoreceptor survival

**Fig 1:**
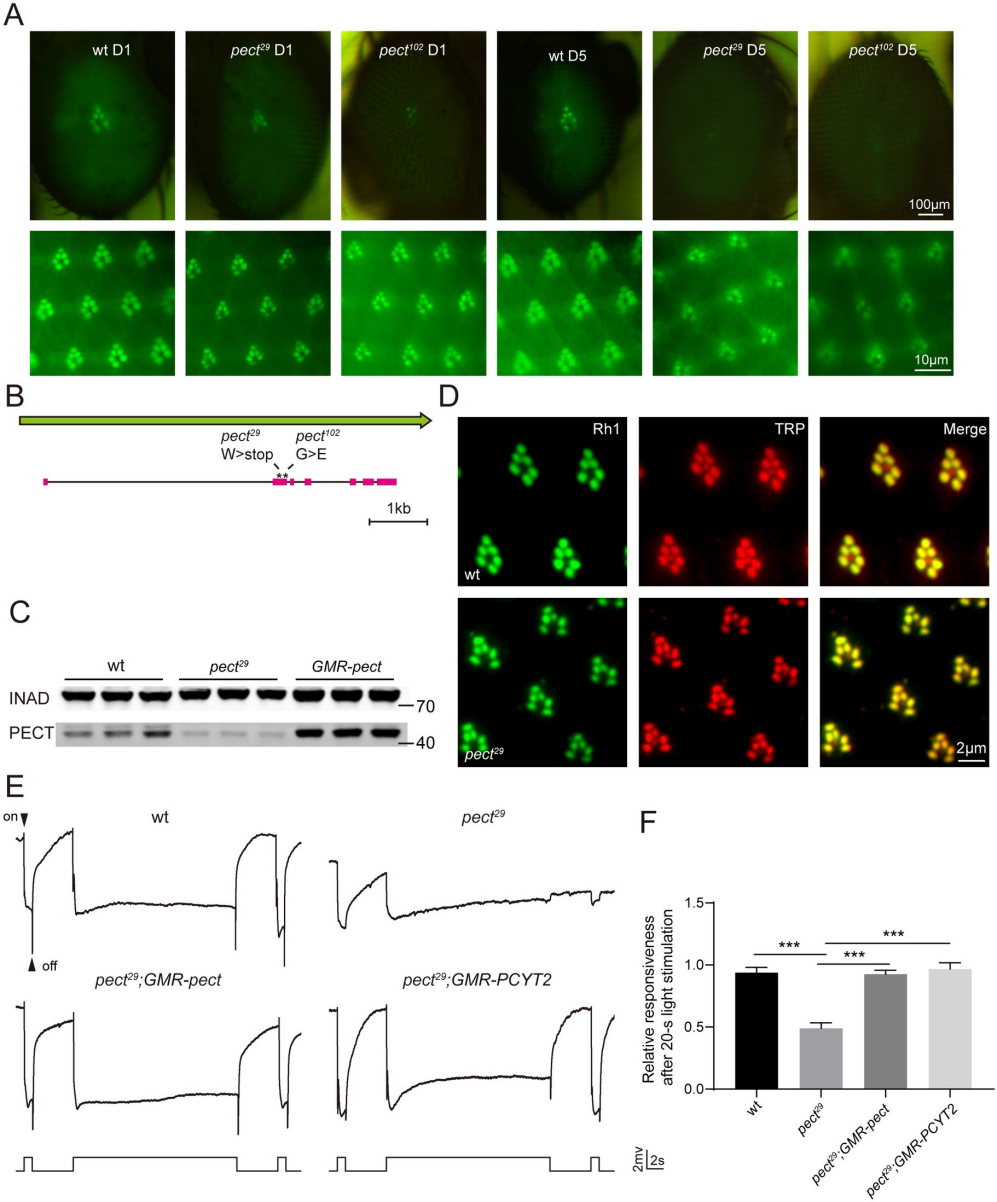
*Pect* mutants displayed prolonged activation of the visual response. (A) Isolation of the *pect*^*29*^ and *pect*^*102*^ mutations via a forward genetic screen. Rh1-GFP fluorescence was detected in the deep pseudopupil (upper panels) and by cornea optical neutralization (lower panel). Images from 1-day-old and 5-day-old wt (wild type, *ey-flp rh1-GFP;FRT40A/GMR-hid CL FRT40A*), *pect*^*29*^ (*ey-flp rh1-GFP;pect*^*29*^ *FRT40A/GMR-hid CL FRT40A*) and *pect*^*102*^ (*ey-flp rh1-GFP;pect*^*102*^ *FRT40A/GMR-hid CL FRT40A*) flies are shown. Scale bars are 100 μm or 10 μm in upper and lower panels, respectively. (B) The *pect* locus and mutations associated with the *pect*^*29*^ and *pect*^*102*^ alleles. (C) Western blot analysis of retinas dissected from 1-d-old wild-type, *pect*^*29*^, and *GMR-pect* flies that were labeled with antibodies against INAD and PECT. (D) Tangential resin-embedded retina sections of compound eyes from ~1-day-old wt, *pect*^*29*^ flies were labeled using antibodies against Rh1 (green) and TRP (red). Scale bar is 2 μm. (E) ERG recordings from 1-day-old wt, *pect*^*29*^, *pect*^*29*^*;GMR-pect* (*ey-flp rh1-GFP;pect*^*29*^ *FRT40A/GMR-hid CL FRT40A;GMR-pect/+*), and *pect*^*29*^*;GMR-PCYT2* (*ey-flp rh1-GFP;pect*^*29*^ *FRT40A/GMR-hid CL FRT40A;GMR-PCYT2/+*) flies. Flies were dark adapted for 2 min and subsequently exposed to a 1-s pulse followed by a 20-s then a 1-s pulse of orange light. On- and off-transients are indicated. (F) ERG amplitudes at the second 1-s pulse are normalized to ERG amplitudes at the first 1-s pulse. Error bars represent SD, and significant differences were determined using the unpaired Student's t-test (*n* = 6, ***p < 0.001).

To identify genes required for photoreceptor survival, we performed EMS mutagenesis and screened chromosomes 2 and 3 using the “*Rh1*::*GFP ey-flp/hid*” system, which generates homozygous mutant eyes and labels photoreceptors with GFP-tagged Rh1 to track cellular integrity [27, 28]. Among the ~50 mutants that exhibited low GFP fluorescence on day 5 but not on day 1, two were lethal mutations in the gene *pect*, which encodes phosphoethanolamine cytidylyltransferase (PECT), a rate-limiting enzyme in the CDP-ethanolamine pathway (Fig 1A) [29, 30]. Both *pect*^*29*^ and *pect*^*102*^ alleles contained a single nucleotide change that affected conserved enzymatic domains (Fig 1B and S1A Fig). Levels of PECT protein were also greatly reduced in *pect*^*29*^ flies (Fig 1C).

We next determined the subcellular localization of PECT by isolating fly heads and separating the cytoplasmic and membrane fractions via subcellular fractionation. PECT immunoreactivity was detected in the cytosol (S2A Fig). We next attempted to use immunohistochemistry to visualize the spatial distribution of PECT within photoreceptors, but the PECT antibody did not work in immunostaining. We therefore expressed Flag-tagged PECT in photoreceptor cells using a promoter associated with the major rhodopsin, *ninaE* (*neither inactivation nor afterpotential E*). PECT protein is largely colocalized with the ER marker calnexin (CNX) [31] (S2B Fig). These data indicate that PECT is a cytosolic protein that localizes to the ER. We then purified PECT recombinant protein and measured its enzymatic activity *in vitro*. CDP-ethanolamine was detected by the mass spectrum after incubating phosphoethanolamine and CTP with PECT, confirming that *Drosophila* PECT is a *bona fide* phosphoethanolamine cytidylyltransferase (S2C and S2D Fig).

### PECT is required for maintaining the visual response

We next asked whether major proteins involved in the phototransduction process were correctly localized in *pect* mutants. Rh1, the major rhodopsin in *Drosophila*, and TRP, the major downstream Ca^2+^/cation channel, localize exclusively to the rhabdomere, tightly-packed microvilli that are critical for phototransduction. Localization patterns for these proteins were the same in wild-type and *pect*^*29*^ flies, indicating that loss of PECT did not affect the formation of the rhabdomere (Fig 1D).

To analyze the visual response of *pect*^*29*^ flies, we performed electroretinogram (ERG) recordings, which measure the summed response of all retinal cells. There are two primary features of the ERG, a sustained corneal negative response, as well as on- and off-transients, which reflect postsynaptic activity in the lamina. In wild-type flies, light induces a rapid corneal negative potential, which quickly returns to baseline after cessation of the 20-s light stimulation. However, light-evoked photoreceptor potential persisted after the 20-s light stimulation, and the ERG transients were diminished in *pect*^*29*^ flies. Expressing wild-type PECT in the compound eyes of *pect*^*29*^ flies via the *GMR* (*glass multiple response*) promoter completely rescued both these ERG phenotypes. Moreover, expressing the mammalian homolog of PECT, *PCYT2*, in *pect*^*29*^ mutant flies also rescued ERG defects (Fig 1E and 1F). These data indicate that PECT activity is required for termination of the visual response.

### Mutations in *pect* lead to light-independent retinal degeneration

**Fig 2:**
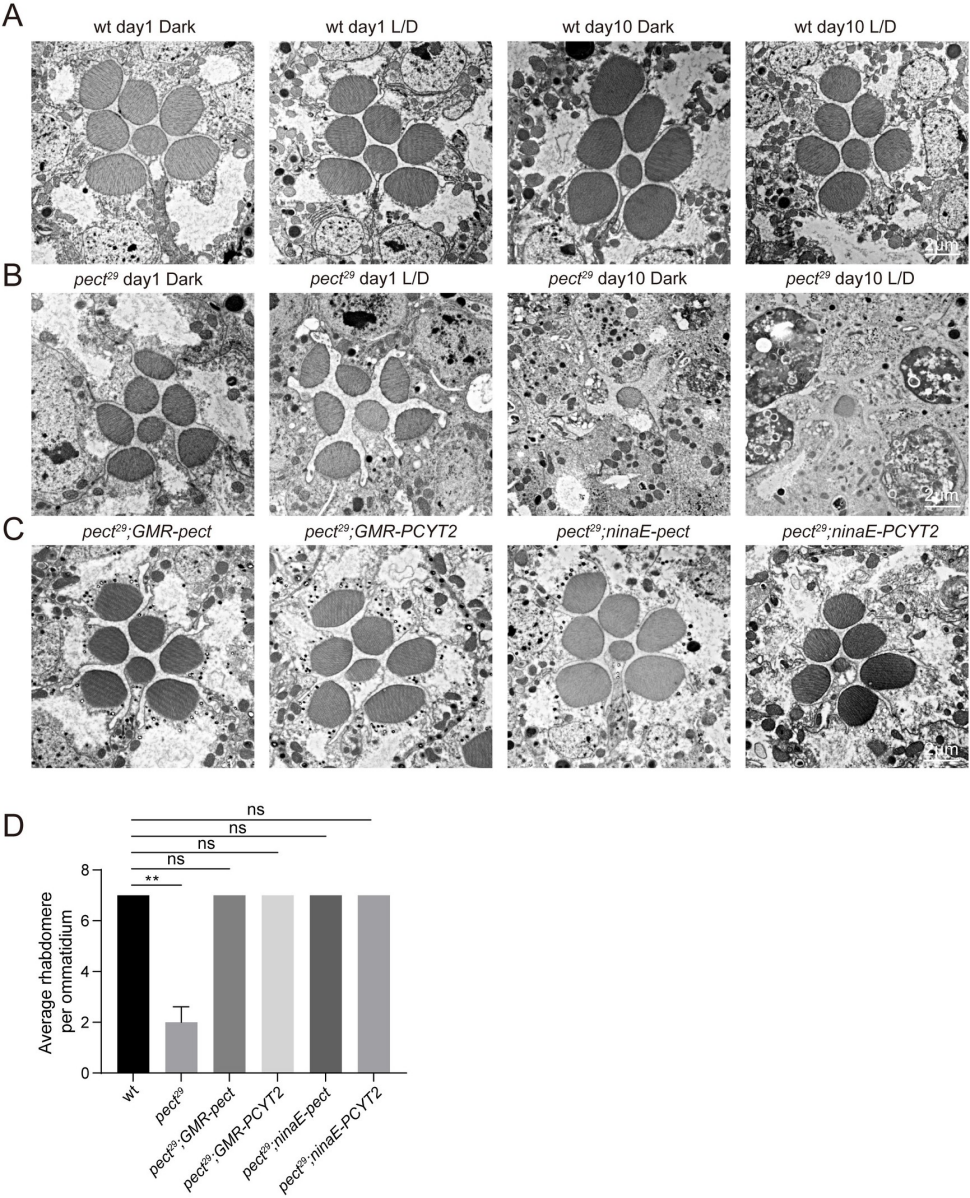
Light-independent retinal degeneration in *pect*^*29*^ flies. (A-B) TEM images of representative ommatidia are shown. Sections were obtained from wild-type (A) or *pect*^*29*^ flies (B), which were raised in the dark or in 12h-light/12h-dark (L/D) cycles for indicated time points. Scale bars are 2 μm. (C) Rescue of retinal degeneration in *pect*^*29*^ mutants by *GMR-pect*, *GMR-PCYT2*, *ninaE-pect*, or *ninaE-PCYT2*. All flies were raised for 10 days under 12h-light/12h-dark cycles. Scale bar is 2 μm. (D) Quantification of rhabdomeres per ommatidium in genotypes indicated. At least 10 ommatidia from each section of three different eyes were quantified for each genotype. All flies were raised for 10 days under 12h-light/12h-dark cycles. Data are presented as mean ± SD, ns, not significant, **p < 0.01 (Student’s unpaired t-test).

We next measured the integrity of photoreceptor cells in *pect*^*29*^ flies using transmission electron microscopy (TEM). In wild-type flies, seven rhabdomeres were detected regardless of fly age and light condition (Fig 2A). However, *pect*^*29*^ flies displayed a gradual loss of photoreceptor cells and rhabdomeres. At 10 days of age, *pect*^*29*^ photoreceptor cells severely degenerated and there was a complete loss of rhabdomeres in R1–R6 cells. This was seen when flies were reared in a constant dark, or a light/dark cycle (Fig 2B). This cell death phenotype was rescued by expressing *pect*, or its mammalian homolog *PCYT2*, via the *GMR* or *ninaE* promoters (Fig 2C and 2D). This also indicates that mutations in *pect* do not cause developmental defects as *ninaE* promoter begins to drive gene expression at the late pupal stage. These results demonstrate that PECT activity is required for photoreceptor cell survival.

### Defects in visual response and loss of photoreceptors seen in *pect* mutants do not result from alternations in DAG and PI metabolism

**Fig 3:**
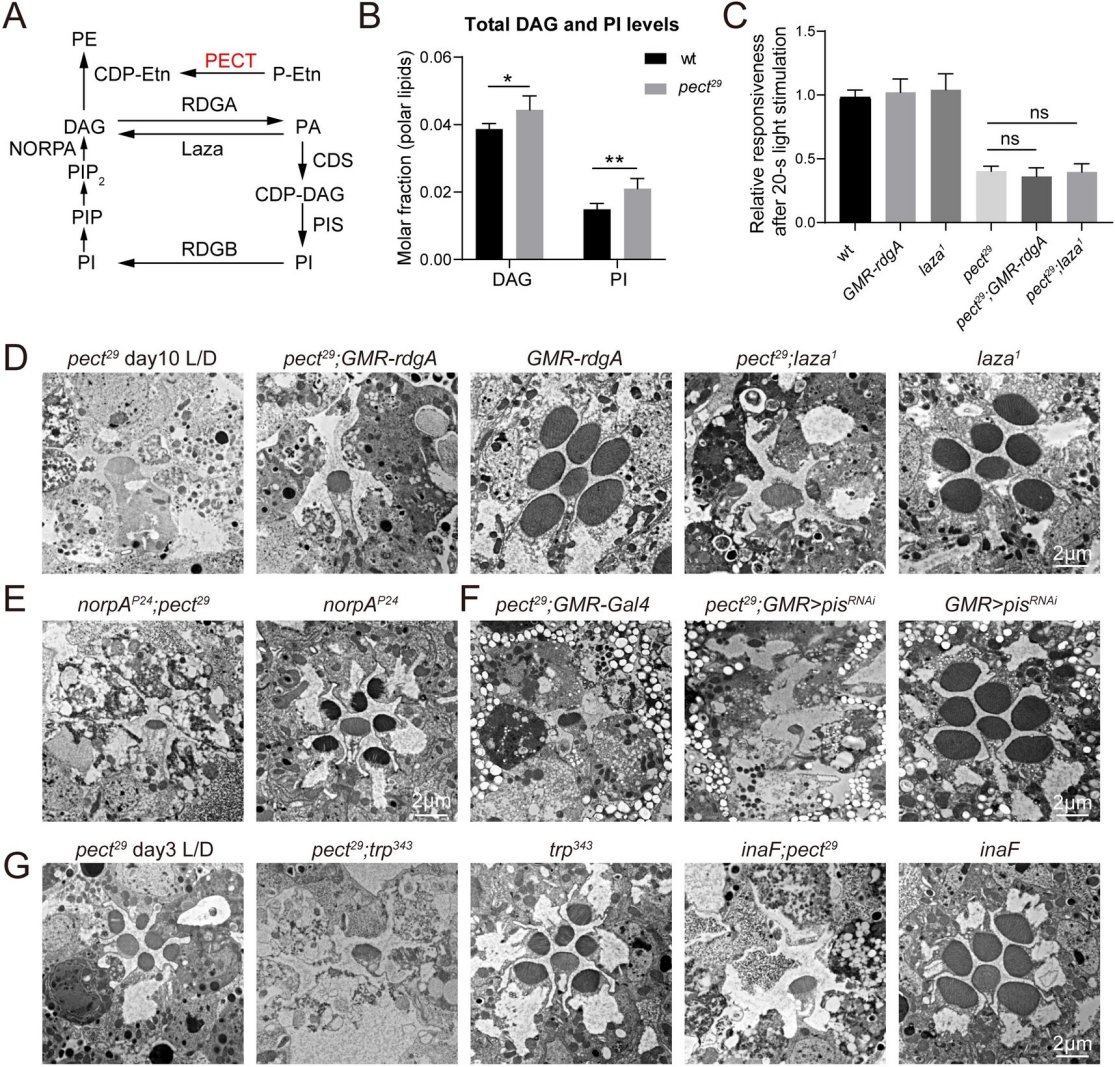
Alteration of PI and DAG levels had no effects on defective photoresponse and retinal degeneration in *pect* mutant flies. (A) Schematic view of the PIP_2_ regeneration cycle. PIP_2_ is hydrolyzed to IP3 and DAG through actions of NORPA. The DAG is converted into PIP_2_ through multistep processes that involve RDGA, CDS, and PIS. DAG can be generated by the dephosphorylation of PA through Laza. PE is synthesized from DAG and CDP-ethanolamine (CDP-Etn), and PECT is involved in the CDP-ethanolamine synthesis pathway. (B) Lipidomic analysis of retinal DAG and PI levels in genotypes indicated. Lipid levels expressed in molar fractions are normalized to total polar lipids. Data are presented as mean ± SD, *p < 0.05, **p < 0.01 (Student’s unpaired t-test). n = 6 replicates of 20 retinas per genotype. (C) Genetically reducing DAG levels did not suppress ERG defects in *pect*^*29*^ mutants. ERG amplitudes of wt, *GMR-rdgA*, *laza*^*1*^, *pect*^*29*^, *pect*^*29*^*;GMR-rdgA* (*ey-flp rh1-GFP;pect*^*29*^ *FRT40A/GMR-hid CL FRT40A;GMR-rdgA/+*), and *pect*^*29*^*; laza*^*1*^ (*ey-flp rh1-GFP;pect*^*29*^ *FRT40A/GMR-hid CL FRT40A; laza*^*1*^) at the second 1-s pulse are normalized to ERG amplitudes at the first 1-s pulse. Error bars represent SD, and significant differences were determined using the unpaired Student's t-test (*n* = 6, ns, not significant). (D-E) Retinal degeneration caused by mutations in *pect* was not altered by genetically reducing DAG levels. TEM Sections were obtained from *pect*^*29*^, *pect*^*29*^*;GMR-rdgA*, *GMR-rdgA*, *pect*^*29*^*;laza*^*1*^, *laza*^*1*^, 3-day-old *norpA*^*P24*^*;pect*^*29*^ (*ey-flp norpA*^*P24*^*/Y;pect*^*29*^ *FRT40A/GMR-hid CL FRT40A*), and 3-day-old *norpA*^*P24*^ Flies. All flies were raised for 10 days under 12h-light/12h-dark cycles except where indicated otherwise. Scale bars are 2 μm. (F) Blocking PI synthesis had no effects on retinal degeneration associated with *pect* mutants. TEM Sections were obtained from *pect*^*29*^*;GMR-Gal4* (*ey-flp rh1-GFP;pect*^*29*^ *FRT40A/GMR-hid CL FRT40A;longGMR-Gal4/+*), *pect*^*29*^*;GMR>pis*^*RNAi*^ (*ey-flp rh1-GFP;pect*^*29*^ *FRT40A/GMR-hid CL FRT40A;longGMR-Gal4/UAS-pis*^*RNAi*^), and *GMR>pis*^*RNAi*^ (*longGMR-Gal4/UAS-pis*^*RNAi*^) flies. All flies were raised for 10 days under 12h-light/12h-dark cycles. The knock-down efficiency of *pis*^*RNAi*^ was 81%. Scale bar is 2 μm. (G) Blocking TRP channels did not suppress retinal degeneration in *pect* mutants. Sections were obtained from *pect*^*29*^, *pect*^*29*^*;trp*^*343*^ (*ey-flp rh1-GFP;pect*^*29*^ *FRT40A/GMR-hid CL FRT40A;trp*^*343*^), *trp*^*343*^, *inaF*;*pect*^*29*^ (*ey-flp inaF/Y;pect*^*29*^ *FRT40A/GMR-hid CL FRT40A*) and *inaF* flies. All flies were raised for 3 days under 12h-light/12h-dark cycles. Scale bar is 2 μm.

It is well established that hydrolysis of phosphatidylinositol 4,5-bisphosphate (PIP_2_) to generate inositol-1,4,5-triphosphate (IP3) and diacylglycerol (DAG) by PLC, which is encoded by the *norpA* (*no receptor potential A*) gene, activates the light-sensitive TRP and TRPL channels in *Drosophila* [24]. Further, DAG or its metabolites may play an excitatory role in phototransduction [32–34]. Therefore, DAG must be rapidly degraded to maintain the resting potential of photoreceptor neurons (Fig 3A). DAG levels were increased by 14% in *pect*^*29*^ mutant retinas, leading us to hypothesize that this excess DAG may sustain active phototransduction in *pect* mutant animals (Fig 3B and S1 Data). We tested this idea by genetically reducing DAG levels in *pect*^*29*^ flies.

We first overexpressed the DAG kinase, RDGA (Retinal Degeneration A) to promote phosphorylation of DAG to phosphatidic acid (PA) [35]. This did not suppress the ERG deficits or photoreceptor neurodegeneration seen in *pect*^*29*^ mutants (Fig 3C and 3D and S3A Fig). We next reduced the generation of DAG from PA by introducing mutations in the gene *lazaro* (*laza*), which encodes a photoreceptor PA phosphatase [36, 37]. ERG responses and retinal degeneration phenotypes were indistinguishable between *pect*^*29*^*;laza*^*1*^ and *pect*^*29*^ flies (Fig 3C and 3D and S3A Fig). Finally, we examined *norpA*^*P24*^*;pect*^*29*^ double mutants, and found the same level of retinal degeneration seen in *pect*^*29*^ mutants (Fig 3E).

Total PI levels were increased by 41% in *pect*^*29*^ retina compared with wild-type (Fig 3B and S1 Data). Since PIP_2_ must be rapidly replenished to maintain a sustained light response, we tested whether reducing PI level could suppress the degeneration phenotype of *pect*^*29*^ flies. PI synthase (PIS) catalyzes the final step of the PI regeneration pathway. Knocking down PIS levels via RNAi (*pis*^*RNAi*^) did not affect the severity of retinal degeneration in *pect*^*29*^ mutants (Fig 3F and S8A Fig). We also generated *pect*^*29*^*;trp*^*343*^ and *inaF;pect*^*29*^ double mutants, thereby inactivating the major light-sensitive TRP channels in photoreceptors. Both of these double mutants exhibited levels of retinal degeneration seen in *pect*^*29*^ flies, suggesting that TRP channel activity does not cause retinal degeneration in *pect* mutants (Fig 3G). In summary, these results demonstrate that the accumulation of DAG and PI did not cause ERG deficits or retinal degeneration in *pect* mutant flies.

### Increasing PECT-independent PE synthesis suppresses retinal degeneration in *pect*^*29*^ flies

**Fig 4:**
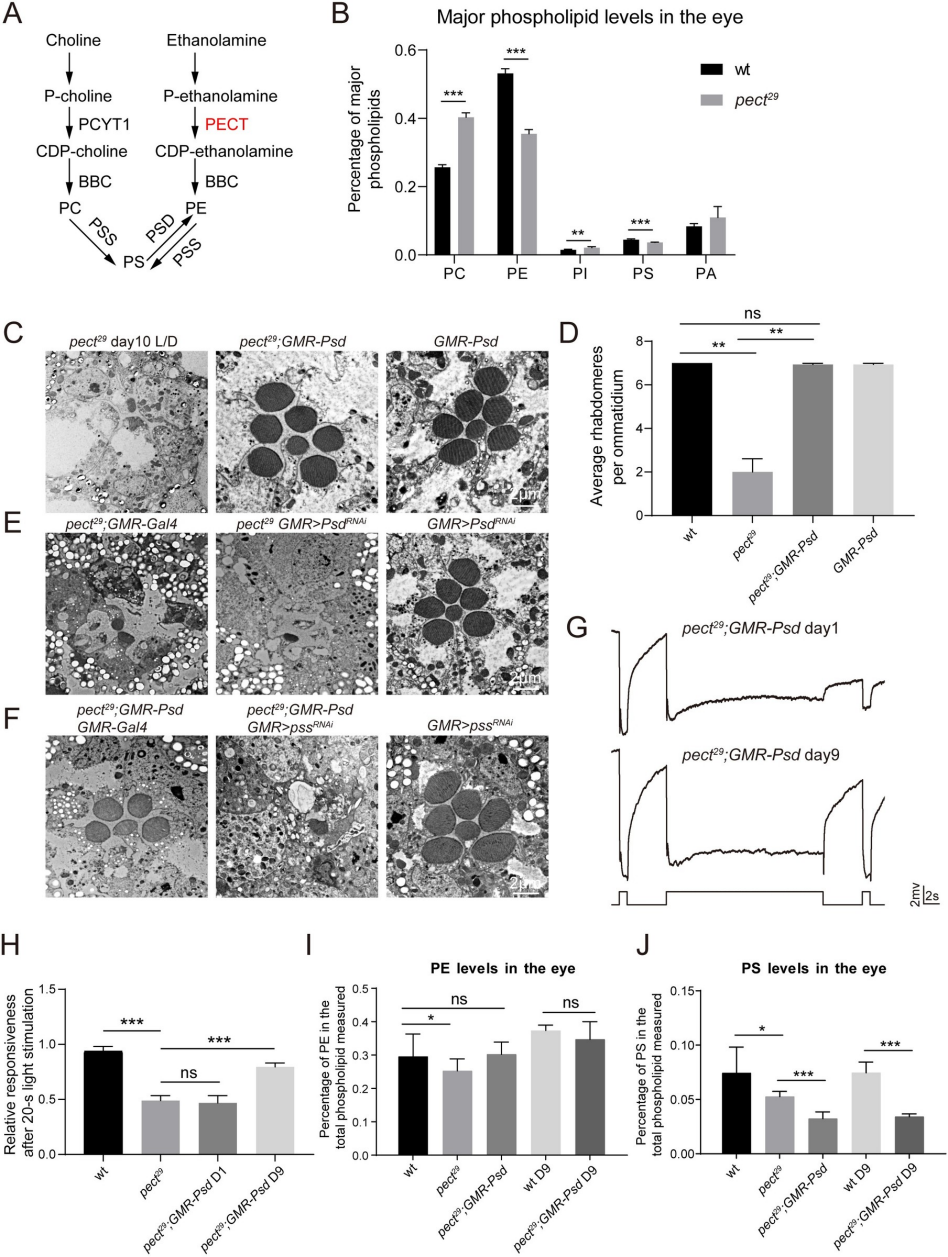
Overexpression of PSD restored the PE levels and suppressed the retinal degeneration and defective photoresponse in *pect*^*29*^ flies. (A) Schematic view of phospholipid metabolism in *Drosophila*. P-choline (Phosphocholine) and P-ethanolamine (Phosphoethanolamine) are converted to CDP-choline and CDP-ethanolamine by PCYT1 or PECT, respectively. PC and PE are generated from CDP-choline or CDP-ethanolamine through actions of BBC. PS is made from PC or PE by base-exchange reactions that are catalyzed by PSS. PE in the mitochondria is generated through decarboxylation of PS by PSD. (B) Lipidomic analysis of major phospholipid levels in retina of wt and *pect*^*29*^ flies. Single phospholipid levels expressed in molar fractions are normalized to total phospholipids. Data are presented as mean ± SD, **p < 0.01, ***p < 0.001 (Student’s unpaired t-test). n = 6 replicates of 20 retinas per genotype. (C) Expressing PSD suppressed retinal degeneration in *pect*^*29*^ flies. Sections were obtained from *pect*^*29*^ (*ey-flp rh1-GFP;pect*^*29*^ *FRT40A/GMR-hid CL FRT40A;GMR-vha68-1/+*), *pect*^*29*^*;GMR-Psd* (*ey-flp rh1-GFP;pect*^*29*^ *FRT40A/GMR-hid CL FRT40A;GMR-Psd/+*), and *GMR-Psd* flies. All flies were raised for 10 days under 12h-light/12h-dark cycles. Scale bar is 2 μm. (D) Quantification of rhabdomeres per ommatidium in genotypes indicated. At least 10 ommatidia from each section of three different eyes were quantified for each genotype. All flies were raised for 10 days under 12h-light/12h-dark cycles. Data are presented as mean ± SD, ns, not significant, **p < 0.01 (Student’s unpaired t-test). (E) The knock-down of *Psd* enhanced the degeneration phenotype in *pect*^*29*^ mutants. Sections were obtained from *pect*^*29*^*;GMR-Gal4*, *pect*^*29*^ *GMR>Psd*^*RNAi*^ (*ey-flp rh1-GFP;pect*^*29*^ *FRT40A UAS-Psd*^*RNAi*^*/GMR-hid CL FRT40A;longGMR-Gal4/+*), and *GMR>Psd*^*RNAi*^ (*UAS-Psd*^*RNAi*^ /*+;longGMR-Gal4/+*). All flies were raised for 5 days under 12h-light/12h-dark cycles. The knock-down efficiency of *Psd*^*RNAi*^ was 45%. Scale bar is 2 μm. (F) Eye-specific knockdown of *pss* in *pect*^*29*^*;GMR-Psd* flies led to severe retinal degeneration. Sections were obtained from *pect*^*29*^*;GMR-Psd GMR-Gal4* (*ey-flp rh1-GFP;pect*^*29*^ *FRT40A/GMR-hid CL FRT40A;GMR-Psd longGMR-Gal4/+*), *pect*^*29*^*;GMR-Psd GMR>pss*^*RNAi*^ (*ey-flp rh1-GFP;pect*^*29*^ *FRT40A/GMR-hid CL FRT40A;GMR-Psd longGMR-Gal4/UAS-pss*^*RNAi*^), and *GMR>pss*^*RNAi*^ (*longGMR-Gal4/UAS-pss*^*RNAi*^). All flies were raised for 10 days under 12h-light/12h-dark cycles. The knock-down efficiency of *pss*^*RNAi*^ was 31%. Scale bar is 2 μm. (G) ERG recordings from 1-day-old and 9-day-old *pect*^*29*^*;GMR-Psd* flies. Flies were dark adapted for 2 min and subsequently exposed to a 1-s pulse followed by a 20-s then a 1-s pulse of orange light. (H) ERG amplitudes at the second 1-s pulse are normalized to ERG amplitudes at the first 1-s pulse. Error bars represent SD, and significant differences were determined using the unpaired Student's t-test (*n* = 6, ns, not significant, ***p < 0.001). (I-J) Lipidomic analysis of retinal PE (I) and PS (J) levels in genotypes indicated. PE and PS levels expressed in molar fractions are normalized to total phospholipids. Data are presented as mean ± SD, ns, not significant, *p < 0.05, ***p < 0.001 (Student’s unpaired t-test). n = 5 replicates of 12 retinas per genotype.

Two major pathways synthesize phosphatidylethanolamine (PE) in the cell, the PECT-dependent CDP-ethanolamine pathway, or through phosphatidylserine (PS) decarboxylation catalyzed by the mitochondrial phosphatidylserine decarboxylase (PSD) (Fig 4A). The synthesis of phosphatidylcholine (PC) and PE involves a series of similar reactions. PS is made from PC or PE by base-exchange reactions that are catalyzed by PSS (phosphatidylserine synthase) (Fig 4A). As PC and PE are major components of cellular membranes, we next assessed the relative levels of phospholipids in *pect*^*29*^ flies. Our lipidomic analysis revealed that the relative PC levels were increased by 57%, whereas the relative PE and PS levels were decreased by 33% and 18%, respectively (Fig 4B and S1 Data). Thus, the phospholipid composition was greatly altered in *pect*^*29*^ flies.

We next genetically manipulated PC and PE levels and investigated the contribution of lipid composition to the mutant phenotypes. We used a heterozygous null allele of *Pcyt1*^*179*^ to decrease PC synthesis in *pect*^*29*^ flies, but this did not suppress the retinal degeneration (S3B and S8B Figs). To examine whether overexpressing PC synthesis enzymes could enhance retinal degeneration, we expressed two enzymes, *bbc* (*bb in a boxcar*) and *Pcyt1* (*Phosphocholine cytidylyltransferase 1*), via the *GMR* promoter. Overexpressing PC synthesis enzymes did not further enhance retinal degeneration in *pect*^*29*^ flies (S3C Fig).

Strikingly, when we expressed PSD to generate PE from PS in mitochondria, the retinal degeneration seen in *pect*^*29*^ flies was suppressed (Fig 4C and 4D). In contrast, knocking down *Psd* in photoreceptor cells via three different *Psd*^*RNAi*^ lines enhanced the *pect*^*29*^ degeneration phenotype. Expressing these *Psd*^*RNAi*^ lines alone did not lead to photoreceptor cell death (Fig 4E, S4A and S8C Figs). Moreover, eye-specific knockdown of PS synthase (*pss*) in *pect*^*29*^*;GMR-Psd* flies led to levels of retinal degeneration that were even more severe than those seen in *pect*^*29*^ flies. Retinal morphology was normal in *pss*^*RNAi*^ flies (Fig 4F and S8D Fig). Since PSS is the only PS synthase in *Drosophila*, it is speculated that both PC and PE are the substrates of PSS. According to our results, PC may be the main substrates of PSS at least in the absence of PECT. Thus *pss* knockdown would reduce PS levels, and subsequently reduce the PE levels generated through the PSD pathway. We next asked whether PSD expression could restore visual responses to normal in *pect*^*29*^ flies. Expressing PSD had little effect on ERG responses in 1-day-old *pect*^*2*9^ flies. In 9-day-old *pect*^*29*^*;GMR-Psd* flies, however, the activation and prolonged after-potential ERG responses seen in *pect*^*29*^ flies were rescued; ERG transients were partially rescued (Fig 4G and 4H and S4B Fig). This delayed effect on ERG response may be explained by the low levels of PE synthesized by the PSD pathway, as the exchange of lipids between the ER and mitochondria is a slow process. Finally, the lipidomic analysis revealed that expressing PSD fully restored PE levels in *pect*^*29*^ cells, both on days 1 and 9 (Fig 4I and S2 Data). In contrast, PSD induction further decreased PS levels by 56% in *pect*^*29*^*;GMR-Psd* flies compared with wild type (Fig 4J and S2 Data). We next examined relative levels of specific PE species and found that levels of PE38:1, PE36:4, and PE36:5 were decreased by 54%, 49%, and 39%, respectively. These levels were fully reversed by PSD overexpression (S5A–S5C Fig and S2 Data). These data indicate that PSD overexpression can restore levels of both total PE and specific PE species in *pect* mutant cells, and further demonstrate that reduced levels of PE caused retinal degeneration and ERG defects in *pect*^*29*^ flies.

### Mitochondria distribution and activity are normal in *pect*^*29*^ retinas

**Fig 5:**
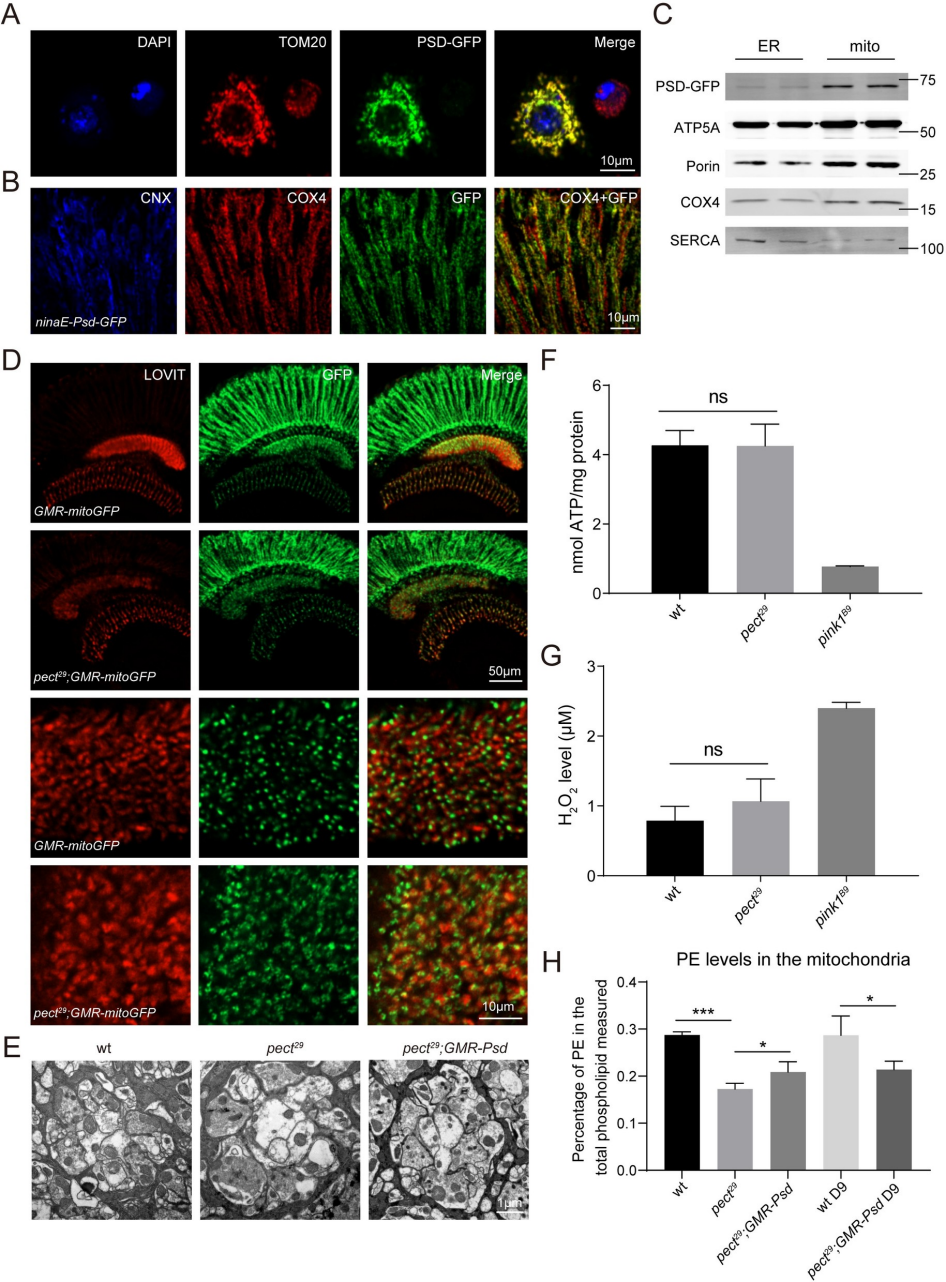
PECT is not required for mitochondria localization or activity. (A-B) Subcellular localization of PSD in (A) S2 cells and (B) adult eyes. (A) S2 cells were transiently transfected with *psd-GFP* and labeled with antibodies against TOM20 (red). Scale bar is 10 μm. (B) Cryostat sections from *ninaE-Psd-GFP* flies were labeled with antibodies against Calnexin (blue), COX4 (red), and GFP (green). Scale bar is 10 μm. (C) Western blot analysis of fractionated ER and mitochondria from *ninaE-Psd-GFP* flies that were labeled with antibodies against GFP, ATP5A, Porin, COX4, and SERCA. (D) Cryostat sections from *GMR-mitoGFP* and *pect*^*29*^*;GMR-mitoGFP* (*ey-flp rh1-GFP;pect*^*29*^ *FRT40A/GMR-hid CL FRT40A;GMR-mitoGFP/+*) flies were labeled with antibodies against LOVIT (red) and GFP (green). The longitudinal views of the visual system were shown in upper panels and the higher magnification of lamina regions were shown in lower panels. Scale bars are 50 μm or 10 μm in upper and lower panels, respectively. (E) TEM micrographs of cartridges containing wild type, *pect*^*29*,^ and *pect*^*29*^*;GMR-Psd* terminals, respectively. Scale bar is 1 μm. (F) Rates of ATP synthesis from the dissected fly retinas of indicated genotypes. Data are presented as mean ± SD, ns, not significant (Student’s unpaired t-test). n = 3. (G) The hydrogen peroxide levels detected in the dissected fly retinas from different genotypes. Data are presented as mean ± SD, ns, not significant (Student’s unpaired t-test). n = 3. (H) Lipidomic analysis of mitochondria isolated from dissected retinas of wt, *pect*^*29*^, and *pect*^*29*^*;GMR-Psd* flies at indicated time points. PE levels expressed in molar fractions are normalized to total phospholipids. Data are presented as mean ± SD, *p < 0.05, ***p < 0.001 (Student’s unpaired t-test). n = 4.

We next sought to determine if PSD localizes exclusively to mitochondria. In S2 cells, PSD-GFP colocalized with TOM20 (Fig 5A). We next generated a transgenic fly that expressed PSD-GFP via the *ninaE* promoter and found that PSD-GFP colocalized with the mitochondrial marker COX4, but not with the ER marker calnexin (CNX) (Fig 5B). Moreover, western bot analysis of ER and mitochondria isolated using a sucrose gradient revealed that PSD expressed in the eye localized exclusively to mitochondria (Fig 5C).

As a mitochondrial protein, PSD may directly modulate mitochondrial PE concentrations. Thus, mitochondrial integrity, which is important for cell survival and for maintaining visual responses, may be disrupted in *pect*^*29*^ mutants [38]. To visualize mitochondria we expressed *mitoGFP* via the *GMR* promoter in *pect*^*29*^ eyes. The pattern of mitochondrial localization and GFP signal was similar between *pect*^*29*^ and control flies (Fig 5D). We next examined the morphology of the lamina cartridge, the organized synaptic modules where R1-R6 photoreceptors project axons to, in *pect*^*29*^ flies. The structure of the lamina cartridge was intact and mitochondria were normal in *pect*^*29*^ flies, although synaptic vesicles were disrupted (Fig 5E) [25]. We compared ATP levels between wild-type and *pect*^*29*^ retinas to assess mitochondrial function and found similar ATP levels in *pect*^*29*^ mutants and controls. In contrast, ATP levels in *pink1*^*B9*^ mutant, a previously characterized mutant which induces mitochondrial impairment [39], were significantly reduced (Fig 5F). We also assessed levels of reactive oxygen species (ROS) by measuring hydrogen peroxide levels but found no differences between *pect*^*29*^ flies and wild type. In contrast, hydrogen peroxide levels were dramatically increased in *pink1*^*B9*^ flies (Fig 5G). Furthermore, we measured mitochondrial transmembrane potential by fluorescent dye TMRM and found no differences between wild-type and *pect*^*29*^ mutant cells (S6A and S6B Fig). Finally, we performed a phospholipid mass spectrometric analysis of isolated mitochondria. Although levels of PE in mitochondria were reduced in *pect*^*29*^ mutant retina, expressing PSD did not restore mitochondrial PE levels in *pect*^*29*^ flies. This was true on day 1 and day 9 (Fig 5H and S3 Data). This may be because PE export from mitochondria is stimulated when substrates for PE production via the CDP-ethanolamine pathway are lacking [14]. Indeed, PSD expression further decreased PS levels. Together, loss of PECT did not affect mitochondrial function, and the disruption of lipid homeostasis in mitochondria did not play a physiological role in *pect*^*29*^ mutant photoreceptor cells.

### ER-mitochondria contacts are required for PE exchange

**Fig 6:**
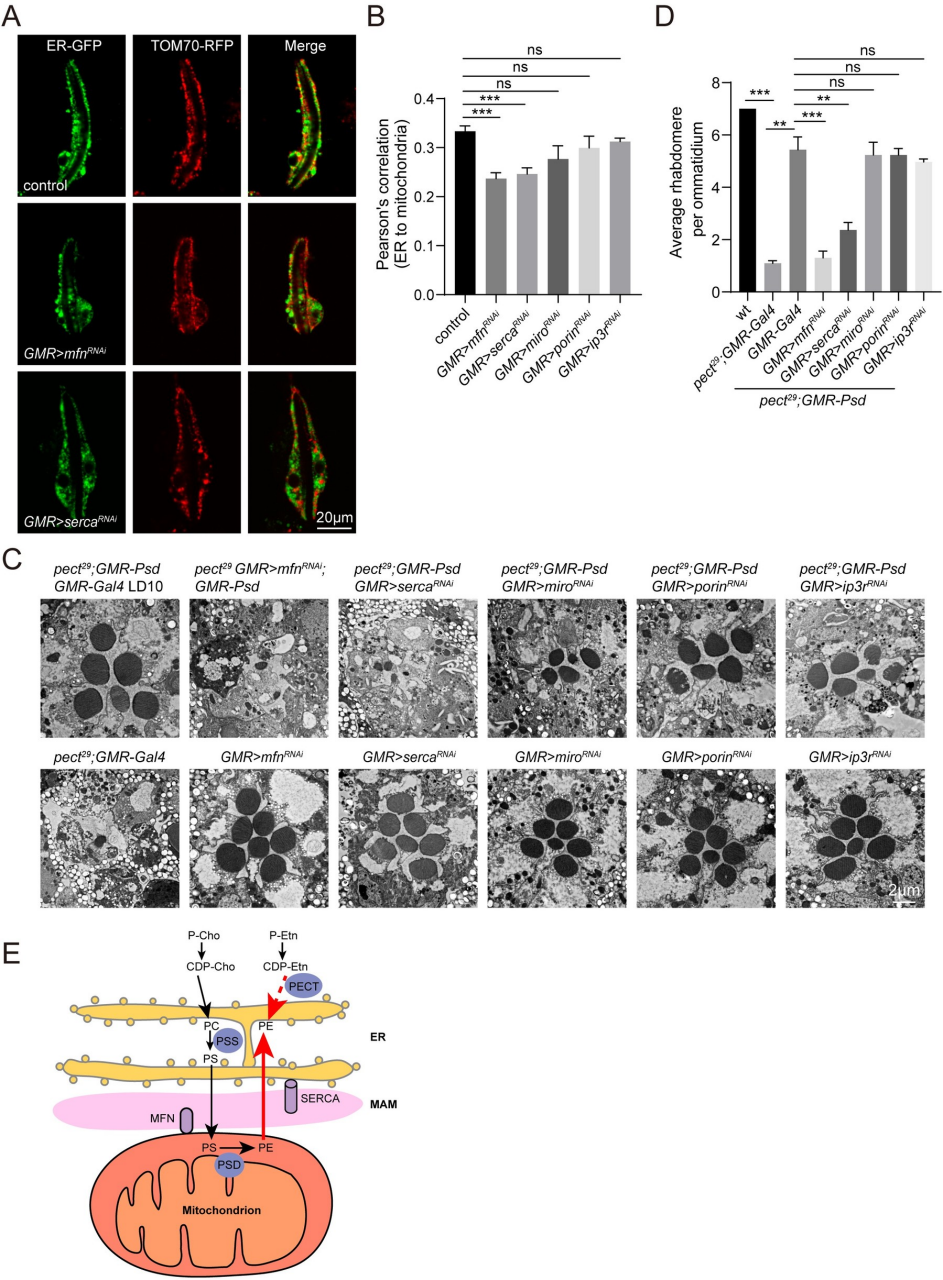
ER-mitochondria contacts are required for the function of PSD in PECT pathway. (A) Live confocal imaging of dissected ommatidia from control (*ninaE-KDEL-GFP/+;trp-Tom70-RFP/+*), *GMR>mfn*^*RNAi*^ (*UAS-mfn*^*RNAi*^*/ninaE-KDEL-GFP;longGMR-Gal4/trp-Tom70-RFP*), and *GMR>serca*^*RNAi*^ (*longGMR-Gal4/ninaE-KDEL-GFP;UAS-serca*^*RNAi*^*/trp-Tom70-RFP*). Scale bar is 20 μm. (B) Quantification of the Pearson’s correlation between ER and mitochondria in genotypes indicated. Three eyes from different flies and at least 20 ommatidia from each eye were quantified for each genotype. Data are presented as mean ± SD, ns, not significant, ***p < 0.001 (Student’s unpaired t-test). (C) TEM sections were obtained from *pect*^*29*^*;GMR-Psd GMR-Gal4* (*ey-flp rh1-GFP;pect*^*29*^ *FRT40A/GMR-hid CL FRT40A;GMR-Psd longGMR-Gal4/+*), *pect*^*29*^ *GMR>mfn*^*RNAi*^*;GMR-Psd* (*ey-flp rh1-GFP;pect*^*29*^ *FRT40A UAS-mfn*^*RNAi*^*/GMR-hid CL FRT40A;GMR-Psd longGMR-Gal4/+*), *pect*^*29*^*;GMR-Psd GMR>serca*^*RNAi*^ (*ey-flp rh1-GFP;pect*^*29*^ *FRT40A/GMR-hid CL FRT40A;GMR-Psd longGMR-Gal4/UAS-serca*^*RNAi*^), *pect*^*29*^*;GMR-Psd GMR>miro*^*RNAi*^ (*ey-flp rh1-GFP;pect*^*29*^ *FRT40A/GMR-hid CL FRT40A;GMR-Psd longGMR-Gal4/UAS-miro*^*RNAi*^), *pect*^*29*^*;GMR-Psd GMR>porin*^*RNAi*^ (*ey-flp rh1-GFP;pect*^*29*^ *FRT40A/GMR-hid CL FRT40A;GMR-Psd longGMR-Gal4/UAS-porin*^*RNAi*^), *pect*^*29*^*;GMR-Psd GMR>ip3r*^*RNAi*^ (*ey-flp rh1-GFP;pect*^*29*^ *FRT40A/GMR-hid CL FRT40A;GMR-Psd longGMR-Gal4/UAS-ip3r*^*RNAi*^), *pect*^*29*^*;GMR-Gal4*, *GMR>mfn*^*RNAi*^ (*UAS-mfn*^*RNAi*^*/+; longGMR-Gal4/+*), *GMR>serca*^*RNAi*^ (*longGMR-Gal4/UAS-serca*^*RNAi*^), *GMR>miro*^*RNAi*^ (*longGMR-Gal4/UAS-miro*^*RNAi*^), *GMR>porin*^*RNAi*^ (*longGMR-Gal4/UAS-porin*^*RNAi*^) and *GMR>ip3r*^*RNAi*^ (*longGMR-Gal4/UAS-ip3r*^*RNAi*^). All flies were raised for 10 days under 12h-light/12h-dark cycles. Scale bar is 2 μm. (D) Quantification of rhabdomeres per ommatidium in genotypes indicated. At least 10 ommatidia from each section of three different eyes were quantified for each genotype. Data are presented as mean ± SD, ns, not significant, **p < 0.01, ***p < 0.001 (Student’s unpaired t-test). The knock-down efficiencies of *mfn*^*RNAi*^, *serca*^*RNAi*^, *miro*^*RNAi*^, *porin*^*RNAi*^, *ip3r*^*RNAi*^ were 54%, 54%, 56%, 48% and 24%, respectively. (E) Model of lipid exchange between ER and mitochondria.PS is generated from PC or PE through PSS which is localized in the MAMs. PS is then transported to the mitochondria inner membrane where PS is converted to PE through PSD. PE synthesized in the mitochondria is transported back to the ER upon cellular PE deficiency.

We next examined the mechanisms by which PSD overexpression suppressed the neurodegeneration and ERG deficits seen in *pect*^*29*^ flies. Since PECT and PSD function to synthesize PE within the ER and mitochondria, respectively, we hypothesized that PE synthesized in mitochondria would be transferred to the ER when cellular PE levels were deficient. Lipid trafficking between the ER and mitochondria occurs at ERMCS [40], thus we tested whether eye-specific knockdown of proteins important for maintaining ERMCS would disrupt the trafficking of PE from mitochondria to the ER. To examine ERMCS *in vivo*, we labeled ER and mitochondria by expressing *KDEL-GFP* and *Tom70-RFP* via the photoreceptor cell-specific *ninaE* and *trp* promoters, respectively. We then knocked down *mfn* (mitofusin), *serca* (*sarco/endoplasmic reticulum Ca*^*2+*^*-ATPase*), *miro* (*mitochondrial Rho*), *porin*, *and ip3r* (*inositol 1*,*4*,*5-triphosphate receptor*), which regulate ERMCS dynamics [41–44], and quantified ERMCS in photoreceptor cells. There were fewer ERMCS in *mfn*^*RNAi*^ and *serca*^*RNAi*^ ommatidia, whereas *miro*^*RNAi*^, *porin*^*RNAi*^, and *ip3r*^*RNAi*^ were not affected. This suggests that the mitochondrial protein MFN, and the ER protein SERCA, are key regulators of ER-mitochondria contacts in photoreceptor cells (Fig 6A and 6B, S7A and S8E–S8I Figs). We next expressed *mfn*^*RNAi*^, *serca*^*RNAi*^, *miro*^*RNAi*^, *porin*^*RNAi*^, and *ip3r*^*RNAi*^ via *GMR-Gal4* in *pect*^*29*^*;GMR-Psd* photoreceptor cells. In agreement with the ERMCS results, both *mfn*^*RNAi*^ and *serca*^*RNAi*^ prevented PSD overexpression from rescuing the retinal degeneration seen in *pect* mutants, *miro*^*RNAi*^, *porin*^*RNAi*^, and *ip3r*^*RNAi*^ had no effect (Fig 6C and 6D). In contrast, knocking down *mfn*, *serca*, *miro*, *porin*, or *ip3r* alone did not affect photoreceptor cells. SERCA is an intracellular calcium pump that transports Ca^2+^ ions from the cytoplasm to the ER lumen and plays an important role in maintaining ER calcium stores. Reduced luminal Ca^2+^ concentration is known to trigger the unfolded protein response (UPR) [45]. To investigate the specific role of SERCA in PE exchange between ER and mitochondria, we performed phospholipid mass spectrum analysis. PSD overexpression increased PE levels and reduced PC levels in *pect*^*29*^ flies. Expressing *serca*^*RNAi*^ significantly decreased PE levels and increased PC levels of *pect*^*29*^*;GMR-Psd* flies, while the PE and PC levels are not changed upon expression of *serca*^*RNAi*^ alone (S7B and S7C Fig and S4 Data). These results suggest that ER-mitochondria contacts are required for the export of PE from mitochondria to ER to maintain cellular PE levels.

## Discussion

In a forward genetic screen to identify genes necessary for photoreceptor cell survival, we isolated mutations in the gene *pect*, which encodes CTP:phosphoethanolamine cytidylyltransferase. In these mutants, light-evoked photoreceptor potentials persisted after 20-s light stimulation, indicating prolonged activation of the visual response. These mutants also exhibited light-independent degeneration of photoreceptor neurons, and lipidomic analysis revealed alterations in major phospholipid composition. We manipulated phospholipid composition via comprehensive genetic interactions and concluded that *pect* mutant phenotypes resulted from PE deficiency. Strikingly, increasing PE synthesis through the PSD pathway effectively suppressed retinal degeneration in *pect* mutants. Finally, the Mitochondria Associated Membrane (MAM)-enriched proteins MFN and SERCA were required for PSD to rescue *pect* phenotypes. We therefore proposed a model in which PE synthesized in the mitochondria through PSD is transported back to the ER through ERMCS when cellular PE is deficient (Fig 6E).

### PE homeostasis is crucial for neuronal survival and activity

Membrane phospholipids, in particular PE, play key roles in regulating neuronal activity and integrity. *Drosophila* photoreceptor neurons utilize the fastest phospholipid signaling cascade and exhibit high rates of membrane trafficking. Therefore, it is particularly important for neurons to maintain phospholipid pools [23, 46]. As phototransduction is completely mediated by phospholipase C (PLC), maintaining levels of PIP_2_ and its product DAG are critical for visual responses [24, 47]. Mutations in the gene *pect* prevent the synthesis of PE from DAG, resulting in increased levels of PI and DAG, and prolonged visual responses. Reducing DAG levels by either introducing mutations in the Lazaro enzyme, which converts PA to DAG, or overexpressing the DAG kinase *rdgA* [36, 37], did not suppress the prolonged afterpotentials in *pect* mutants. Moreover, inhibiting PI synthesis failed to suppress the retinal degeneration and defective ERG responses [48]. In addition, TRP channels were constitutively active in *rdgA* mutant photoreceptors, and the over-activation and retinal degeneration of *rdgA* mutants were rescued in *rdgA;trp* double mutants [34]. In the case of *pect*, loss of TRP channels did not affect the severity of neurodegeneration, indicating that RDGA and PECT function in different pathways. In conclusion, PECT regulates photoreceptor function and morphology independent of PI and DAG metabolism. Recent evidence suggested TRPs are mechanosensitive channels, and membrane physical properties are involved in channel activation [49–51]. Indeed, the rhabdomere size was reduced in the 1-day-old *pect* mutants, indicating changes in the physical properties of the lipid bilayer. We speculate that alterations in phospholipid composition in *pect* mutants may change the compact structure and membrane fluidity of rhabdomere, which leads to prolonged activation of TRP channels.

We further provide direct evidence that maintaining cellular PE levels is critical for neuronal function and integrity. First, disruption of the PSD pathway by knocking down PSD enhanced retinal degeneration in *pect* mutants. More importantly, overexpression of PSD restored cellular PE levels, and thus greatly suppressed both the prolonged afterpotentials in response to light and retinal degeneration of *pect* mutants. Moreover, this suppression was fully reversed by down-regulating PSS. It has been reported that knockdown of *Psd* resulted in light-dependent retinal degeneration by preventing autophagy-dependent rhodopsin degradation since the abundance of PE could positively regulate autophagy [26, 52]. However, for *pect* mutants, rhodopsin turn-over is normal and degeneration is independent of light and TRP channel activity, suggesting that rhodopsin homeostasis does not cause *pect*-induced cell death. Furthermore, the light-independent retinal degeneration in *pect* mutants suggested that cellular PE homeostasis independently contributes to maintaining neuronal activity and integrity.

### Complementary roles of CDP-ethanolamine and the PSD pathway in PE synthesis

The majority of mitochondrial PE is synthesized *in situ* in mitochondria via PSD. In contrast, only a small fraction of mitochondrial PE is made in the ER by the CDP-ethanolamine pathway [14, 15, 53, 54]. Maintaining mitochondrial PE levels is critical for mitochondrial respiratory capacity, morphology, and distribution, thus deletion of PSD impairs mitochondrial function, resulting in lethality [6, 55–57]. The *pect* mutant photoreceptor cells are dysfunctional, and both cellular and mitochondrial levels of PE are significantly reduced. Although it has been reported that < 30% depletion of mitochondrial PE by RNAi silencing of PSD alters mitochondrial morphology and function in mammalian cells [5], ~40% reduction in mitochondrial PE levels in *pect*^*29*^ flies did not affect mitochondrial activity, morphology, or axonal localization. Overexpression of PSD, which completely rescued photoreceptor function and integrity in *pect* mutants, restored total PE levels but not levels of mitochondrial PE. Therefore, disruption of the CDP-ethanolamine PE synthesis pathway did not impair mitochondrial function. Disrupting PE synthesis through the CDP-ethanolamine pathway does not result in a mitochondrial phenotype because mitochondrial PE is synthesized locally through PSD, although the CDP-ethanolamine pathway does contribute to total mitochondrial PE levels.

A recent study reported that yeast Psd1 localizes to both mitochondria and the ER through its transmembrane region (TMR) [58]. This forced us to consider that PSD in the ER may have restored cellular PE homeostasis in *pect* mutants. However, *Drosophila* PSD lacks the TMR sequence required for Psd1 to localize to the ER, and PSD remains in mitochondria when overexpression. ER-mitochondria connections are necessary for the efficient exchange of phospholipids between organelles [40, 59]. Several proteins, including the mitofusin MFN2 and the ER Ca^2+^ ATPase SERCA, have been implicated in maintaining ERMCS, thus facilitating phospholipid exchange. Consistent with previous reports, we found that the fly proteins, MFN and SERCA, stabilize ER-mitochondrial contact sites [60]. Importantly, disrupting ER-mitochondria contacts through *mfn*^*RNAi*^ or *serca*^*RNAi*^ completely blocked the ability of PSD to rescue *pect* mutant phenotypes. This is also consistent with a recent study that described an unexpected role of MFN2 in PS transfer between the ER and mitochondria [61]. However, as loss of SERCA induces UPR reaction, we cannot rule out the possible role of inducing UPR in disruption of PE homeostasis. Taken together, these analyses prove that cellular PE homeostasis is maintained, in part, by synthesizing PE in mitochondria and then exporting this PE to cellular pools. Thus, mitochondria play a critical role in cellular phospholipid homeostasis.

### Similar PE species are generated by the CDP-ethanolamine and PSD pathways

PE species generated from the CDP-ethanolamine and PSD pathways are different, especially fatty acids in the *sn-2* position, where PE from the CDP-ethanolamine and PSD pathways prefer mono-/di-unsaturated and polyunsaturated fatty acids, respectively [18]. It has been suggested that individual molecular species of PE may play specialized roles in cellular signaling, which explained why deletion of either *Pisd* or *Pcyt2* causes embryonic lethality in mice [6, 12, 62]. Here we saw that as total PE levels decreased, the proportion of most PE species were unaffected. In contrast, the PE species PE38:1, PE36:4, and PE36:5 were down-regulated, and PSD overexpression fully restored levels of these three PE species, as well as total PE levels. This suggests that the same PE species are generated by the CDP-ethanolamine and PSD pathway *in vivo*, although they might prefer different substrates *in vitro*. These data further showed that PE generated in the mitochondria can compensate for cellular PE deficiency in the *Drosophila* visual system.

A recent study also identified recessive lethal mutations in *pect* and demonstrated that the biosynthesis of specific phospholipids is linked to neurodegeneration and synaptic vesicle loss in adult *Drosophila* photoreceptors [25]. In *pect*^*omb593*^ mutants levels of PE 34:1 and PE 36:2 were reduced, but the overall proportions of PE species were not significantly changed, whereas we detected a significant reduction in total cellular PE levels. The different results of phospholipid composition in *pect* mutants may come from the different alleles analyzed. In contrast to the nonsense *pect*^*29*^ mutation, the *pect*^*omb593*^ contains a hypomorphic mutation with a single amino acid change (H55Y). Thus PECT^H55Y^ might reduce PE levels to a much lesser extent than complete loss of *pect*, and this cellular PE reduction could be compensated by the alternative PSD pathway. Moreover, knocking down *srebp* (regulatory element binding protein) reversed the loss of synaptic vesicles, but failed to suppress axonal or retinal degeneration in *pect*^*omb593*^ mutants [25]. Therefore, SREBP is a downstream factor of PE deficiency and specifically functions in maintaining the synaptic vesicle pools. Here, we demonstrated that increasing PE levels through induction of the PSD pathway restored the cellular PE levels and effectively suppressed retinal degeneration and defective phototransduction in *pect* mutants, supporting disruption of PE homeostasis is the major cause of photoreceptor cell degeneration and loss of synaptic transmission.

### Physiological functions of both PE synthesis pathways

Phospholipid composition is critical for cellular homeostasis, and alterations in the composition of major phospholipid PE are implicated in multiple diseases [61, 63]. Cellular PC/PE molar ratios can influence energy metabolism in numerous organelles and thus lead to disease conditions such as steatohepatitis, obesity, and muscular dystrophy [63–65]. Cellular PC levels were elevated by 57% in *pect*^*29*^ retinas compared with wild type, but inhibiting PC synthesis did not suppress the retinal degeneration, suggesting that PE levels, but not the PC/PE ratio, are crucial for neuronal function.

Genetic mutations that affect PE synthesis have been identified in several human autosomal-recessive disorders. In particular, mutations in PISD, the human counterpart of fly PSD, cause Liberfarb syndrome, which is a multisystem disorder affecting the eyes, ears, bone, and brain [22]. Studies in patient-derived fibroblasts revealed impaired phospholipid metabolism, altered mitochondrial respiration, and fragmentation of the mitochondrial network [20, 21]. Recently, mutations in PCYT2, the human counterpart of fly PECT, have been associated with hereditary spastic paraplegia. Lipidomic analysis of patient fibroblasts revealed profound lipid abnormalities impacting both neutral etherlipid and etherphospholipid metabolism [7]. As mutations in fly *pect* affect phospholipid composition, resulting in defective photoresponse and severe retinal degeneration, this system represents a conserved model for studying diseases associated with PE deficiency. Moreover, our data provide direct genetic evidence that induction of mitochondrial PE synthesis can compensate for deficiencies in cellular PE, and suggest that mitochondrial phospholipid synthesis and trafficking represent a promising therapeutic target for treating disorders associated with defective phospholipid composition.

## Materials and methods

### Fly stocks

The following stocks were obtained from the Bloomington Stock Center: (1) w^*^;laza^*1*^, (2) *norpA*^*P24*^, (3) w^*^;trp^*343*^, (4) *inaF*^*P106x*^, (5) w^*^;Pcyt1^*179*^ *h*^*1*^ *P[neoFRT] 80B/TM3*,*Ser*^*1*^, (6) *y*^*1*^ *v*^*1*^*;P[TRiP*.*JF03315]attP2* (*pis*^*RNAi*^), (7) *y*^*1*^ sc^*^ *v*^*1*^ *sev*^*21*^*;P[TRiP*.*HMS05791]attP40* (*Psd*^*RNAi*^), (8) *y*^*1*^ sc^*^ *v*^*1*^ *sev*^*21*^*;P[TRiP*.*HMC03883]attP40* (*mfn*^*RNAi*^), (9) *y*^*1*^ sc^*^ *v*^*1*^ *sev*^*21*^*;P[TRiP*.*HMS02878]attP2* (*serca*^*RNAi*^), (10) *y*^*1*^ sc^*^ *v*^*1*^ *sev*^*21*^*;P[TRiP*.*GL01583]attP2* (*miro*^*RNAi*^), (11) *y*^*1*^ *v*^*1*^*;P[TRiP*.*JF03251]attP2/TM3*,*Sb*^*1*^ (*porin*^*RNAi*^), (12) *y*^*1*^ *v*^*1*^*; P[TRiP*.*JF01957]attP2* (*ip3r*^*RNAi*^), (13) *y*^*1*^ sc^*^ *v*^*1*^ *sev*^*21*^*; P[VALIUM20-EGFP*.*shRNA*.*4]attP2*, (14) w^*^;P[longGMR-GAL4]2, (15) *y*^*1*^ w^*^; wg^*Sp-1*^*/CyO; P[longGMR-GAL4]3/TM2*, (16) *M(vas-int*.*Dm)ZH-2A;M(3xP3-RFP*.*attP)ZH-86Fb*, (17) *w*^*1118*^. The *GMR-mitoGFP*, *ninaE-KDEL-GFP*, *trp-Tom70-RFP*, *ey-flp ninaE-Rh1-GFP;FRT40A*, and *ey-flp ninaE-Rh1-GFP;GMR-hid CL FRT40A /CyO* flies were maintained in the laboratory of T. Wang. Flies were maintained in 12hr light/12hr dark cycles with 2000 lux illumination at 25°C, unless different conditions were described in the text.

### EMS mutagenesis

The second chromosome of *ey-flp*,*ninaE-Rh1-GFP;FTR40A* flies was isogenized, and young male flies were fed with 25 mM EMS (ethyl methanesulfonate) (Sigma) in 2% sucrose for 8 h. Mutagenized flies were mated immediately to *ey-flp ninaE-Rh1-GFP;GMR-hid CL FRT40A /Cyo* flies. F1 progenies were screened by performing the fluorescence deep pseudopupil (DPP) assay at days 1 and 5 following eclosion, as described [27]. Approximately 100,000 F1 flies were screened.

### Generation of transgenic flies

The *pect*, *rdgA*, *Pcyt1*, and *Psd* cDNAs were amplified from the cDNA clones GH23180, RH08828, LD46058, and LD21713, respectively, which were obtained from the *Drosophila* Genomic Resource Center. The *bbc* cDNA was amplified from the *w*^*1118*^ cDNAs. The *PCYT2* cDNA was amplified from the cDNA clone IOH3644, which was obtained from the *Ultimate ORF Clones* (Invitrogen). To express *pect* or *PCYT2* cDNAs under control of the *ninaE* or *GMR* promoter [27], the cDNAs were subcloned into the *pninaE-attB* or *pGMR-attB* vector between the *EcoRI* and *NotI* sites. To express *rdgA*, *Pcyt1*, *bbc*, or *Psd* cDNAs under control of the *GMR* promoter, the cDNAs were subcloned into the *pGMR-attB* vector between the *NotI* and *XbaI* sites. The *pect* cDNA was subcloned into the *pIB-c-3xflag* vector between the *EcoRI* and *NotI* sites. The fragment *pect-3xflag* was subcloned into the *pninaE-attB* vector between the *EcoRI* and *XbaI* sites. The *Psd* cDNA was subcloned into the *pIB-c-GFP* vector between the *Acc65I* and *NotI* sites. The fragment *Psd-GFP* was subcloned into the *pninaE-attB* vector between the *Acc65I* and *XbaI* sites. These constructs were injected into *M(vas-int*.*Dm)ZH-2A;M(3xP3-RFP*.*attP)ZH-86Fb* embryos and transformants were identified based on eye color. The *3xP3-RFP* marker was eliminated by crossing to a Cre-expressing line.

To generate *pss*^*RNAi*^, *Psd*^*RNAi3*^, and *Psd*^*RNAi4*^ flies, the following 21-nt sequences were used: *pss*^*shRNA*^: 5’-GGAGCATATTCTACTGGATTG-3’, *Psd*^*shRNA3*^: 5’- GCAGCCCATTAAAGGACCACA-3’, *Psd*^*shRNA4*^: 5’-GCTCAGATTCGGCGCAATATA-3’. Annealed oligo pairs were cloned into the VALIUM20 vector at the *NheI* and *EcoRI* sites [66]. Constructs were injected into *M(vas-int*.*Dm)ZH-2A;M(3xP3-RFP*.*attP)ZH-86Fb* embryos and transformants were identified based on eye color.

### Quantitative real-time PCR

RNAi efficiencies of RNAi flies driven by *GMR-Gal4* were analyzed by real-time polymerase chain reaction (qPCR). RNA was isolated from dissected adult fly retinas using TRIzol (Invitrogen). Total RNA was treated with TURBO DNase (ThermoFisher) and 500 ng RNA was subjected to reverse transcription using the PrimeScript RT Master Mix (Takara). Quantitative PCR was performed using TB Green *Premix Ex Taq* II (Takara), and results were analyzed with a CFX96 Real-Time PCR Detection System (Bio-Rad). Primers used for qPCR are as follows: *pss* forward primer: 5’-ATGAAGAAGCGCACTAATTCACG-3’; *pss* reverse primer: 5’-CCTGATTTGTAGGCGGGATG-3’; *pis* forward primer: 5’-GCCGAGCACGATAACGTCTT-3’; *pis* reverse primer: 5’-GGACATGAACCAGAAGGCGA-3’; *serca* forward primer: 5’-ATGACCATGGCTCTGTCCG-3’; *serca* reverse primer: 5’-CTTCTGCAGACAACGGTGTC-3’; *mfn* forward primer: 5’-GAGACGACCACCTTTATCAACG-3’; *mfn* reverse primer: 5’-GCCACCTTCATGTGATCCCG-3’; *miro* forward primer: 5’-ACAAGTACAAGTTGCTGCC-3’; *miro* reverse primer: 5’-CTGTGCTTGTAGAACAGTAT-3’; *porin* forward primer: 5’-CTGTCCAGGATCAGCTCCTC-3’; *porin* reverse primer: 5’-CTTGACGTTCTCATGGCCGT-3’; *Pcyt1* forward primer: 5’-TCAAGCGGAAGTACGTCCC-3’; *Pcyt1* reverse primer: 5’-TCCTCGTCGTGAGAGTAGG-3’; *ip3r* forward primer: 5’-ATGGGCGACAATATAATTGGCTC-3’; *ip3r* reverse primer: 5’-CTGAACTTCTTAGGCGGACAG-3’; *RPL32* forward primer: 5’-CAGTCGGATCGATATGCTAAGC-3’; *RPL32* reverse primer: 5’-AATCTCCTTGCGCTTCTTGG-3’.

### Generation of antibodies directed against PECT

The *pect* cDNA was subcloned into the *pRSETB* vector between the *BglII* and *EcoRI* sites, and the plasmid was transformed into BL21 (DE3) competent cells. Protein expression was induced with 0.5 mM IPTG, and cells were harvested and resuspended in phosphate-buffered saline (PBS, pH 7.4) supplemented with complete EDTA-free protease inhibitor cocktail (Sigma). Cells were lysed by freeze-thaw followed by sonication, and inclusion bodies were collected by centrifugation for 10 min, 13000 rpm at 4°C. The pellet was washed with washing buffer 1 (PBS, 1 mM EDTA, 1% Triton X-100), followed by washing buffer 2 (PBS, 1 mM EDTA, 2 M urea). The final pellet was homogenized in SDS sample buffer and fractionated by SDS-PAGE. The band corresponding to the size of PECT was sliced and further sent to immunize a rabbit.

### Generation of antibodies directed against CNX and Porin

Polyclonal antibodies against *Drosophila* Calnexin 99A (CNX) were generated by immunizing a rabbit with a synthetic peptide (CESREPAQTEESNTKTRKRQARKEK) conjugated to KLH. Polyclonal antibodies against *Drosophila* Porin were generated by immunizing a rat with a synthetic peptide (CGYQTAFDTQQSKLTTNNF) conjugated to KLH.

### Subcellular fractionation

To separate the cytoplasmic and membranal fractions, subcellular fractionation was performed, as described [67]. Twenty fly heads were collected and homogenized in lysis buffer (10 mM Tris-HCl pH 7.5, 150 mM NaCl, 0.5 mM EDTA) containing complete EDTA-free protease inhibitor cocktail (Sigma). Homogenates were centrifuged at 950 *g* at 4°C for 10 min to remove cell debris. The supernatant was centrifuged at 21,000 *g* at 4°C for 40 min. The supernatant and pellet fractions were solubilized in SDS sample buffer to equal volumes for western blot analysis. For characterization of subcellular fractions, the following antibodies were used: Tubulin (mouse, 1:2000, Developmental Studies Hybridoma Bank), Rh1 (mouse, 1:2000, Developmental Studies Hybridoma Bank), and PECT (rabbit, 1:2000).

ER and mitochondria fractions were isolated from fly heads, as described [68]. Briefly, 1,000 fly heads were homogenized with MTE (270 mM mannitol, 10 mM Tris, 0.1 mM EDTA, pH 7.4) containing complete EDTA-free protease inhibitor cocktail (Sigma). The homogenate was centrifuged twice at 600 *g* for 5 min to remove large debris and nuclei. The supernatant was centrifuged for 10 min at 15,000 *g* at 4°C to separate crude ER and crude mitochondria. The supernatant containing crude ER was loaded on 1.3 M/1.5 M/2.0 M step sucrose density gradients and centrifuged at 152,000 *g* for 70 min. The large band at the interface of the 1.3M sucrose gradient layer was collected with a syringe, diluted with MTE, and centrifuged at 126,000 *g* for 45 min. The final pellet was resuspended in PBS and stored at -20°C. The mitochondria pellet was washed with MTE three times and resuspended in MTE buffer. The crude mitochondria were loaded on 1.7 M/1.0 M step sucrose density gradients and centrifuged at 40,000 *g* for 22 min. The band at the interface of the 1.7 M and 1.0 M sucrose layers was extracted with a syringe, diluted with MTE, and centrifuged at 15,000 *g* for 10 min. The final pellet was resuspended in PBS and stored at -20°C. For characterization of subcellular fractions, the following antibodies were used: GFP (rabbit, 1:2000, Torrey Pines Biolabs), ATP5A (mouse, 1:2000, #ab14748, Abcam), Porin (rat, 1:500), COX4 (rabbit, 1:2000) [69], and SERCA (rabbit, 1:1000, Santa Cruz).

### PECT enzyme activity assays

To purify PECT recombinant protein, the *pect* cDNA was subcloned into the *pMAL* vector between the *EcoRI* and *SalI* sites. The plasmid was transformed into BL21 (DE3) competent cells and grown at 37°C in LB medium containing Ampicillin and 0.2% glucose to an OD_600_ of around 0.8. Protein expression was induced with 0.1 mM IPTG and culture incubated for 24 h at 13°C, 220 rpm. Cells were harvested and resuspended in column Buffer (20 mM Tris-HCl, 200 mM NaCl, 1 mM EDTA, pH 7.4) supplemented with complete EDTA-free protease inhibitor cocktail (Sigma). Cells were lysed by freeze-thaw followed by sonication. The supernatant was collected by centrifugation for 30 min, 20,000 *g* at 4°C. Then it was diluted 1:6 with Column Buffer and loaded onto an amylose column (New England Biolabs). The fusion protein was eluted with column buffer supplemented with 10 mM maltose.

The purified MBP-PECT or MBP proteins were used for enzyme activity assay, as described [70]. The reaction mixture contained the following components in 25 μl: 20 mM Tris-HCl (pH 7.7), 10 mM MgCl_2_, 2 mM CTP, 1 mM phosphoethanolamine, 1 mM DTT, and 1 μM MBP-PECT or MBP. All reactions were incubated at 37°C for 15 min followed by boiling for 3 min to inactivate the enzyme. The product CDP-ethanolamine was analyzed with liquid chromatography-mass spectrometry (LC-MS). The Dionex Ultimate 3000 UPLC system was coupled to a TSQ Quantum Ultra triple-quadrupole mass spectrometer (ThermoFisher, Waltham, MA), equipped with a heated electrospray ionization (HESI) probe operated in negative ion mode. The reaction mixture was separated by an Atlantis Silica column (2.1 × 100 mm, 3 μm, Waters, Milford, MA). Separations were performed using binary gradient mobile phases, consisting of water with 0.1% formic acid (A) and ACN with 0.1% formic acid (B). Data were acquired in selected reaction monitoring (SRM) for CDP-ethanolamine with a transition of 447.100/324.148. Data analysis and quantification were performed using software Xcalibur 3.0.63 (ThermoFisher, Waltham, MA).

### Electroretinogram recordings

ERG recordings were performed as described [71]. Two glass microelectrodes filled with Ringer’s solution were inserted into small drops of electrode cream (PARKER LABORATORIES) that were placed onto the surface of the compound eye and the thorax. A Newport light projector (model 765) was used for stimulation. ERG signals were amplified with a Warner electrometer IE-210 and recorded with a MacLab/4s analog-to-digital converter and the Clampex 10.2 program (Molecular Devices, San Jose, CA). All recordings were carried out at room temperature.

### Immunohistochemistry

Whole-mount immunolabeling was performed to locate PECT-FLAG in adult flies. Hemisected fly heads were fixed with 4% paraformaldehyde (PFA) in PBS for 1 h on ice. Dissected retinas were incubated with primary antibodies against FLAG (mouse, 1:200, Sigma), and CNX (rabbit, 1:200). Secondary antibodies against mouse Alexa Fluor 647 and rabbit Alexa Fluor 568 were used (1:400, Invitrogen).

To investigate the localization pattern of major proteins involved in the phototransduction process, resin-embedded sections were labeled as described [72]. Hemisected fly heads were fixed with 4% PFA in PBS and embedded in LR White resin. Cross sections (0.5 μm) of compound eyes were cut through the distal region of the retina, which included the R7 cells. Sections were incubated with primary antibodies against Rh1 (mouse monoclonal 4C5, 1:200, Developmental Studies Hybridoma Bank) and TRP (rabbit, 1:200) [71] at room temperature for 1 h. Secondary antibodies against mouse Alexa Fluor 488 and rabbit Alexa Fluor 568 were used (1:400, Invitrogen).

To visualize PSD-GFP and mitochondria localization patterns, cryo-stat sections were prepared and labeled as described [27]. Briefly, fly heads were fixed with 4% PFA in PBS (pH 7.4) for 1 h on ice. Fixed heads were infiltrated with 12% sucrose overnight at 4°C, embedded in O.C.T (Sakura), and sectioned at 10-μm thickness. Cryosections were immunolabeled with primary antibodies against Cnx99A (mouse, 1:50, Developmental Studies Hybridoma Bank), COX4 (rabbit, 1:100) [69], or LOVIT (rat, 1:100) [73]. Secondary antibodies against mouse Alexa Fluor 647, rabbit Alexa Fluor 568, and rat Alexa Fluor 568 were used (1:400, Invitrogen). Images were captured with a Nikon A1-R confocal microscope (Nikon, Tokyo, Japan). Acquired images were processed using Photoshop CC2017 and NIS-Elements AR Analysis 5.20.00.

### Cell transfection

S2 cells were transfected by adding 2.5 μg plasmid mixed with 1 μl VigoFect (Vigorous Biotechnology) into the cell media. Twenty-four hours following transfection, cells were suspended and adhered to a poly-lysine coated coverslip for 1 h and then fixed with 4% PFA in PBS for 30 min at room temperature. Cells were incubated with primary antibodies against Tom20 (rat, 1:100) [74] and GFP (rabbit, 1:200, Invitrogen). Secondary antibodies against rat Alexa Fluor 568 and rabbit Alexa Fluor 488 were used (1:400, Invitrogen). Images were captured with a Nikon A1-R confocal microscope (Nikon, Tokyo, Japan). Acquired images were processed using Photoshop CC 2017 and NIS-Elements AR Analysis 5.20.00.

### Mitochondria activity assays

The ATP assay kit was obtained from Beyotime and the assay was performed according to the manufacturer’s instruction. To measure total ATP level, 10 dissected retinas were homogenized in extraction buffer. After centrifugation to remove cell debris, the supernatant was added to the substrate solution. The luminescence was recorded in an Illuminometer (EnSpire Multimode Plate Reader; PerkinElmer, Waltham, MA).

The intracellular hydrogen peroxide assay kit was obtained from Sigma and the assay was performed according to the manufacturer’s instruction. Briefly, 10 dissected retinas were homogenized in assay buffer. The homogenized supernatant was mixed with a fluorescent peroxide sensor and incubated at room temperature for 15 min. The fluorescence intensity at (λex = 490/λem = 525 nm) was measured in a fluorescence plate reader (EnSpire Multimode Plate Reader; PerkinElmer, Waltham, MA).

To check mitochondrial membrane potential, *pect*^*29*^ *FRT40A/CyO* flies were crossed with *hs-flp;FRT40A ubi-GFP* flies, and heat shocked at the 1^st^ instar larva stage at 37°C for 1 hour to induce mosaic clone. Pupa eyes were then dissected at 36~40 h APF (after pupa formation), and stained with 100 nM TMRM (tetramethylrhodamine methyl ester, ThermoFisher). GFP positive cells are quantified as wild-type cells, and GFP negative cells are *pect*^*29*^ mutant cells.

### Single ommatidium observation

Ommatidia from 1-day-old flies were dissected in Schneider’s *Drosophila* medium (Invitrogen) as describe [75]. Images were taken within 30 min under a Nikon A1-R confocal microscope (Nikon, Tokyo, Japan). The Pearson’s Coefficient, as a measure of colocalization between KDEL-GFP and Tom70-RFP, was calculated using NIS-Elements Advanced Research Analysis 5.20.00 (Nikon, Tokyo, Japan).

### Transmission electron microscopy

To visualize *Drosophila* retina ultrastructure, adult fly heads were dissected, fixed, dehydrated, and embedded in LR White resin (Electron Microscopy Sciences) as described [67]. Thin sections (80 nm) prepared at a depth of 30–40 μm were stained with uranyl acetate and lead citrate (Ted Pella) and examined using a JEM-1400 transmission electron microscope (JEOL, Tokyo, Japan) equipped with a Gatan CCD (4k × 3.7k pixels, USA).

TEM of photoreceptor terminals was performed as described [73]. Adult fly heads were dissected in 4% PFA and the retinas were removed. The dissected lamina was fixed in a solution with 4% PFA and 2.5% glutaraldehyde for 2 h on ice, followed by fixation in 1% osmium tetroxide for 1.5 h at 4°C. Tissues were then dehydrated in a series of ethanol dilutions at 4°C (10-min wash in 10, 25, 40, 55, 70, 85, 95, and 30-min wash in 100% ethanol for 5 times). Samples were gradually infiltrated with 2 ratios of ethanol and Eponate 12 (Ted Pella), finally going into 3 changes of pure resin. Samples were allowed to infiltrate in pure resin overnight on a rotator and embedded in Eponate 12 resin (Ted Pella). Thin sections (80 nm) were stained with uranyl acetate and lead-citrate (Ted Pella) and examined using a JEM-1400 transmission electron microscope (JEOL, Tokyo, Japan) equipped with a Gatan CCD (4k × 3.7k pixels, USA).

### Lipid extraction and mass spectrometric analysis

Lipids were extracted from retinas or mitochondria as described [76]. Mitochondria were extracted from dissected *Drosophila* retinas using a mitochondria isolation kit (Abcam) designated for tissue extraction according to the manufacturer's protocol, following tissue homogenization using a Dounce homogenizer (KONTES).

Polar lipids were analyzed on an Exion UPLC system coupled with a triple quadrupole/ion trap mass spectrometer (QTRAP 6500 PLUS, Sciex). PC-d_31_ (16:0/18:1), PE-d_31_ (16:0/18:1), PS-d_31_ (16:0/18:1), PI-d_31_ (16:0/18:1), PA-d_31_ (16:0/18:1), PA (17:0/17:0), PG-d_31_ (16:0/18:1), LPC-17:0, LPE-17:1, LPS-17:1, LPI-17:1 were obtained from Avanti Polar Lipids (Alabaster, AL, USA). Glycerol lipids triacylglycerides (TAG) and diacyglycerides (DAG) were analyzed using a modified protocol of reverse-phase HPLC/ ESI/MS described previously [77]. DAG species were quantified using 4ME 16:0 Diether DG as an internal standard (Avanti Polar Lipids, Alabaster, AL, USA). Separation of individual lipid classes of polar lipids by NP-HPLC was carried out using a Phenomenex Luna 3 μ-silica column (i.d. 150 × 2.0 mm) with the following conditions: mobile phase A (chloroform: methanol: ammonium hydroxide, 89.5:10:0.5) and mobile phase B (chloroform: methanol: ammonium hydroxide: water, 55:39:0.5:5.5). The initial mobile phase proportion was 95% (A) / 5% (B), which was held for 5 min. A was then linearly decreased to 60% in 7 min, which was held for 4 min. A was further decreased to 30%, which was held for 15 min. Finally, the initial mobile phase conditions were restored and held for 5 min. Data were acquired in multiple reaction monitoring (MRM) in a combined workflow for polar lipids analysis. The source parameters are as follows: curtain gas: 20, ion spray voltage: 5500 V, temperature: 400°C, ion source gas 1: 35, ion source gas 2: 35. MS profiles were recorded under both positive and negative modes in separate runs (resolution 60,000), and mass accuracy of less than 2 ppm was obtained throughout the analytical runs. The mole fraction of each lipid was normalized to the mole fraction of total polar lipids.

### Statistical analysis

All data in bar and line graphs are expressed as Means ± SDs. Significant differences between different groups were determined using Student’s unpaired t-tests in Graphpad Prism 6 (Graphpad Software Inc.). (***p < 0.001; **p < 0.01; *p < 0.05; ns, not significant).

## Supporting information

S1 Fig The protein sequence containing the point mutations of *pect*^*29*^ and *pect*^*102*^ is highly conserved. (A) The amino acid sequence of mammalian PCYT2 and *Drosophila* PECT are shown. Identical residues are enclosed in black boxes. The running tally of amino acids is indicated to the right. The mutated amino acids in *pect*^*29*^ and *pect*^*102*^ alleles are indicated with red asterisks.
(TIF)

S2 Fig PECT localizes in the ER and catalyzes the synthesis of CDP-Etn from P-Etn and CTP *in vitro*. (A) The cytoplasmic (c) and membranal (m) fractions from wild-type and *GMR-pect* fly head extracts were separated. Western blots were probed with antibodies against PECT, Rh1, and Tubulin. (B) Eyes from *ninaE-pect-3xflag* flies were labeled with antibodies against FLAG (green) and Calnexin (red). Scale bar is 10 μm. (C-D) Mass-spectrum analysis of PECT enzyme activity. (C) There is no characteristic peak of CDP-Etn in the reaction mixed with a negative control MBP. (D) The product CDP-Etn was detected by the mass spectrum after incubated with PECT recombinant protein.
(TIF)

S3 Fig Genetic manipulation of PC did not affect retinal degeneration of *pect*^*29*^. (A) ERG recordings from 1-day-old *pect*^*29*^*;laza*^*1*^, *laza*^*1*^, *pect*^*29*^*;GMR-rdgA*, *GMR-rdgA* flies. Flies were dark-adapted for 2 min and subsequently exposed to a 1-s pulse followed by a 20-s then a 1-s pulse of orange light. (B) Reducing PC level did not suppress retinal degeneration in *pect*^*29*^ flies. TEM sections were obtained from wt, *pect*^*29*^, *pect*^*29*^*;Pcyt1*^*179*^*/+ (ey-flp rh1-GFP;pect*^*29*^ *FRT40A/GMR-hid CL FRT40A;Pcyt1*^*179*^*/+)*, and *Pcyt1*^*179*^*/+* flies. The *Pcyt1* mRNA expression level was reduced to 53% in *Pcyt1*^*179*^*/+* flies. Scale bar is 2 μm. (C) Overexpressing PC synthesis enzymes did not further enhance retinal degeneration. TEM sections were obtained from *pect*^*29*^*;GMR-bbc (ey-flp rh1-GFP;pect*^*29*^ *FRT40A/GMR-hid CL FRT40A;GMR-bbc/+)*, *GMR-bbc*, *pect*^*29*^*;GMR-Pcyt1 (ey-flp rh1-GFP;pect*^*29*^ *FRT40A/GMR-hid CL FRT40A;GMR-Pcyt1/+)*, and *GMR-Pcyt1* flies. Scale bar is 2 μm.
(TIF)

S4 Fig PSD knockdown enhanced the degeneration phenotype in *pect*^*29*^ mutants, while PSD overexpression partially rescued the ERG transients in *pect*^*29*^ mutants. (A) The knockdown of *psd* enhanced the degeneration phenotype in *pect*^*29*^ mutants. Sections were obtained from *pect*^*29*^*;GMR-Gal4*, *pect*^*29*^*;GMR>Psd*^*RNAi3*^ (*ey-flp rh1-GFP;pect*^*29*^ *FRT40A/GMR-hid CL FRT40A;longGMR-Gal4/UAS-Psd*^*RNAi3*^), *pect*^*29*^*;GMR>Psd*^*RNAi4*^ (*ey-flp rh1-GFP;pect*^*29*^ *FRT40A/GMR-hid CL FRT40A;longGMR-Gal4/UAS-Psd*^*RNAi4*^), *GMR>Psd*^*RNAi3*^ (*longGMR-Gal4/UAS-Psd*^*RNAi3*^) and *GMR>Psd*^*RNAi4*^ (*longGMR-Gal4/UAS-Psd*^*RNAi4*^). All flies were raised for 5 days under 12h-light/12h-dark cycles. Scale bar is 2 μm. (B) Quantification of off-transients in different genotypes. One third of *pect*^*29*^*;GMR-Psd* flies display normal off transients on day 9.
(TIF)

S5 Fig PSD overexpression restored levels of individual PE species in *pect*^*29*^ mutant retinas. (A-C) Lipidomics analysis of specific PE species levels in genotypes indicated. Specific PE species levels expressed in molar fractions are normalized to total PE levels. Data are presented as mean ± SD, *p < 0.05, ***p < 0.001 (Student’s unpaired t-test). n = 5 replicates of 12 retinas per genotype.
(TIF)

S6 Fig The mitochondrial transmembrane potential was not changed in *pect*^*29*^ mutant cells. (A) Pupae eyes staining of *pect*^*29*^ mosaic clones from *hs-flp;pect*^*29*^ *FRT40A/FRT40A ubi-GFP* flies with 100 nM TMRM for 15 min at room temperature, followed by live confocal imaging immediately. GFP negative cells are *pect*^*29*^ mutant cells. Scale bar is 50 μm. (B) Quantification of relative TMRM fluorescence intensity in wild-type and *pect*^*29*^ mutant cells. Data are presented as mean ± SD. ns, not significant (Student’s unpaired t-test). Four different pupae eyes were used for quantification.
(TIF)

S7 Fig SERCA is required for PE exchange between ER and mitochondria. (A) ER-mitochondria contacts are not affected by *miro*^*RNAi*^, *porin*^*RNAi*,^ and *ip3r*^*RNAi*^. Live confocal imaging of dissected ommatidia from control (*ninaE-KDEL-GFP/+;trp-Tom70-RFP/+*), *GMR>miro*^*RNAi*^ (*longGMR-Gal4/ninaE-KDEL-GFP;UAS-miro*^*RNAi*^*/trp-Tom70-RFP*), *GMR>porin*^*RNAi*^ (*longGMR-Gal4/ninaE-KDEL-GFP;UAS-porin*^*RNAi*^*/trp-Tom70-RFP*) and *GMR>ip3r*^*RNAi*^ (*longGMR-Gal4/ninaE-KDEL-GFP;UAS-ip3r*^*RNAi*^*/trp-Tom70-RFP*). Scale bar is 20 μm. (B-C) Lipidomic analysis of retinal PE (B) and PC (C) levels in genotypes indicated. PE and PC levels in molar fractions are normalized to total phospholipids. Data are presented as mean ± SD from 5 replicates of 20 retinas per genotype, ***p < 0.001 (Student’s unpaired t-test).
(TIF)

S8 Fig Relative knockdown efficiencies of all RNAi lines used. (A-I) RNAi efficiency was determined by using quantitative Real Time PCR (qPCR). Total RNA was extracted from the dissected fly retina of indicated genotypes. The relative expression of target genes was normalized to *RPL32*, which serves as an internal control. Data are presented as mean ± SD, *p < 0.05, **p < 0.01 (Student’s unpaired t-test). n = 3.
(TIF)

S1 Data Lipidomic analysis measuring lipid levels in *pect*^*29*^ mutant retinas. Percentage of membrane lipids measured in wild type and *pect*^*29*^ mutant retinas at day1.
(XLSX)

S2 Data Lipidomic analysis measuring cellular phospholipid levels in *pect*^*29*^ and *pect*^*29*^*;GMR-Psd* fly retinas. Percentage of cellular phospholipids measured in wild type, *pect*^*29*^ and *pect*^*29*^*;GMR-Psd* fly retinas at day1 and day9.
(XLSX)

S3 Data Lipidomic analysis of mitochondrial phospholipid levels in *pect*^*29*^ and *pect*^*29*^*;GMR-Psd* fly retinas. Percentage of mitochondrial phospholipids measured in wild type, *pect*^*29*^ and *pect*^*29*^*;GMR-Psd* fly retinas at day1 and day9.
(XLSX)

S4 Data Lipidomic analysis of cellular phospholipid levels in *pect*^*29*^, *pect*^*29*^*;GMR-Psd* and *pect*^*29*^*;GMR-Psd/GMR>serca*^*RNAi*^ fly retinas. Percentage of cellular phospholipids measured in *FRT40A;GMR-Gal4*, *pect*^*29*^*;GMR-Gal4*, *pect*^*29*^*;GMR-Psd/GMR-Gal4*, *pect*^*29*^*;GMR-Psd/GMR>serca*^*RNAi*^ and *GMR>serca*^*RNAi*^ fly retinas at day1.
(XLSX)
