# Supplementary figures and images for: PE homeostasis rebalanced through mitochondria-ER lipid exchange prevents retinal degeneration in Drosophila
Source: PLoS Genet. 2020 Oct 16;16(10):e1009070. doi: 10.1371/journal.pgen.1009070 (PMC7592913; doi:10.1371/journal.pgen.1009070)

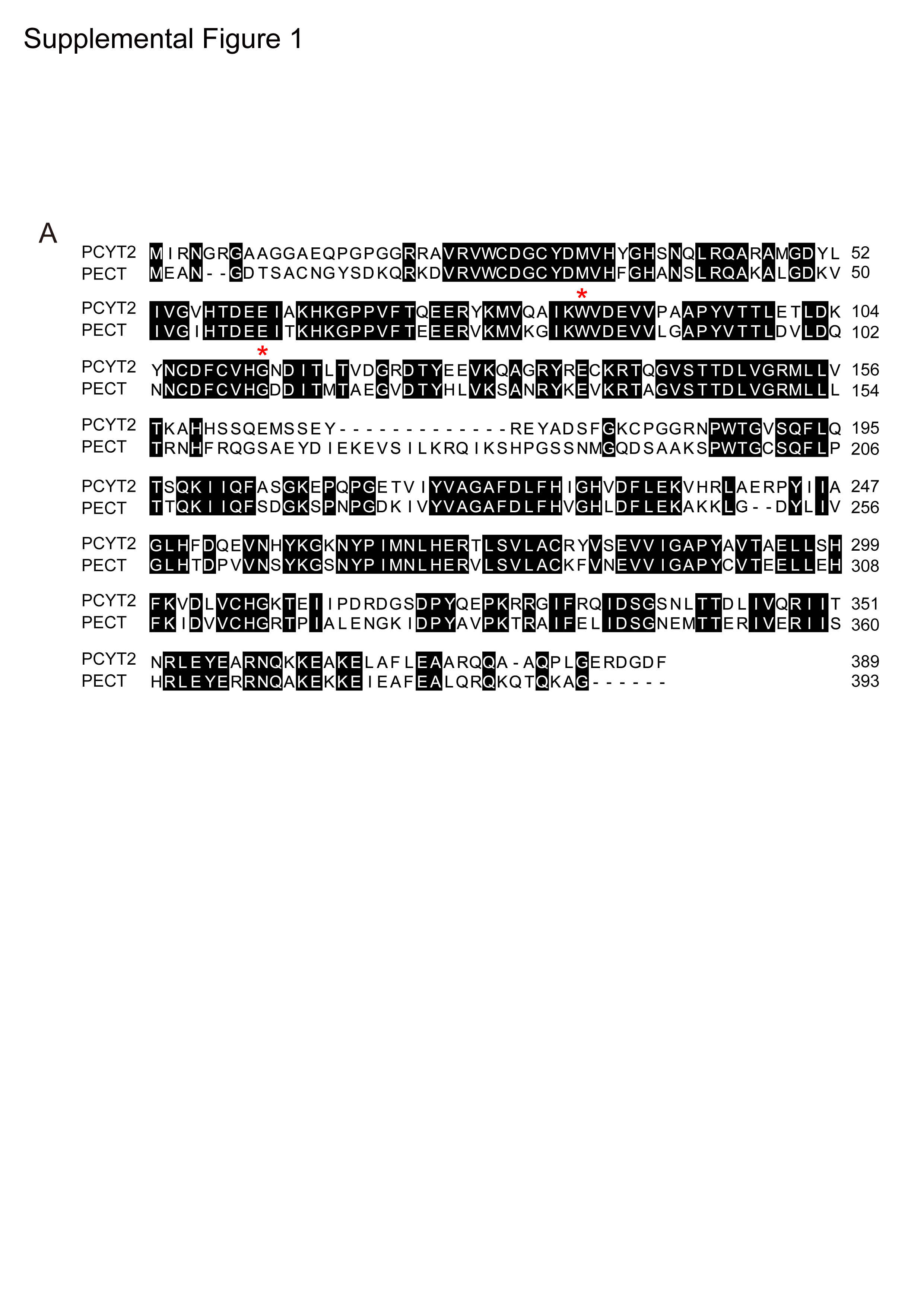

Supplement: S1 Fig — (A) The amino acid sequence of mammalian PCYT2 and Drosophila PECT are shown. Identical residues are enclosed in black boxes. The running tally of amino acids is indicated to the right. The mutated amino acids in pect29 and pect102 alleles are indicated with red asterisks. (TIF) [file pgen.1009070.s001.tif]

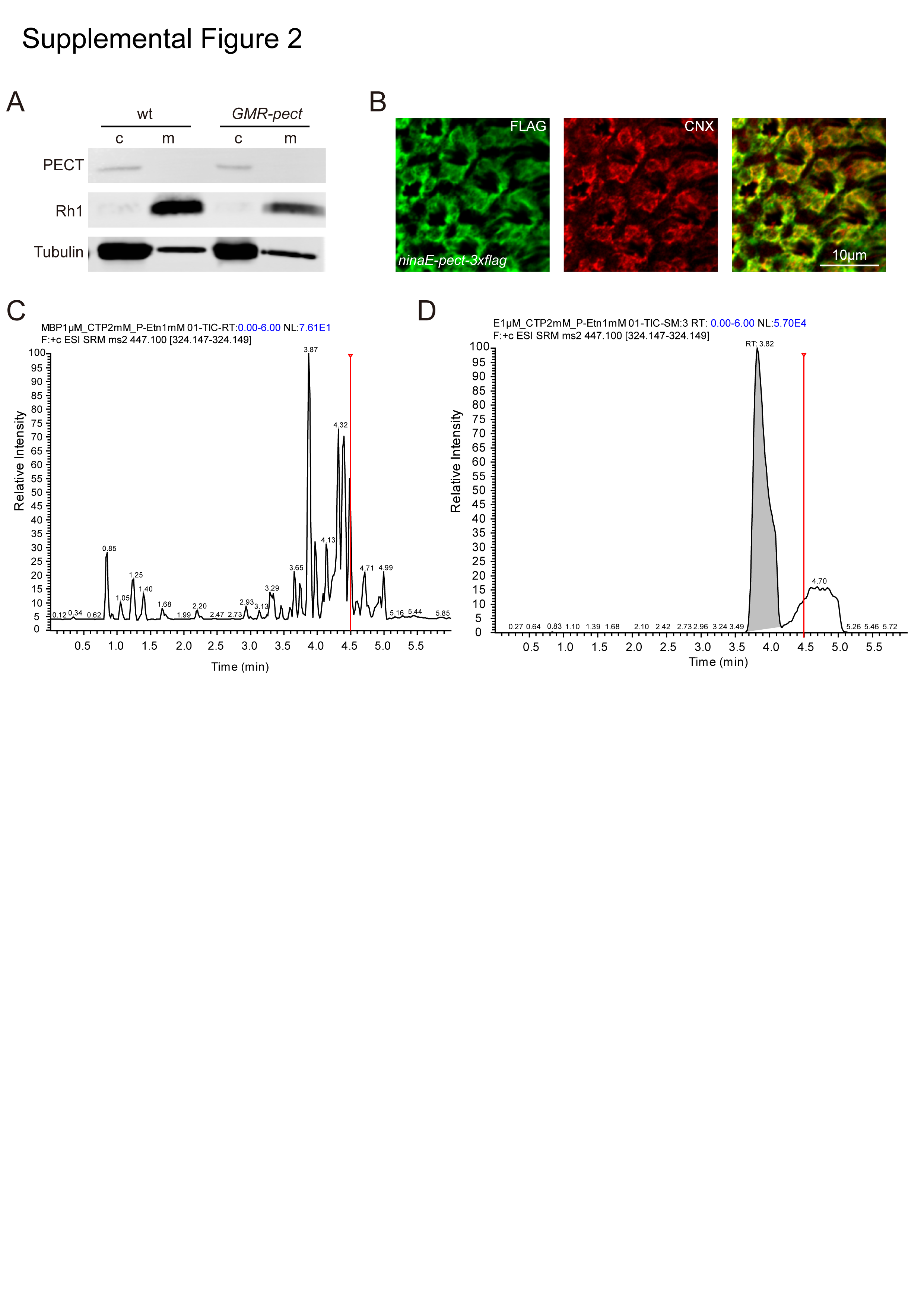

Supplement: S2 Fig — (A) The cytoplasmic (c) and membranal (m) fractions from wild-type and GMR-pect fly head extracts were separated. Western blots were probed with antibodies against PECT, Rh1, and Tubulin. (B) Eyes from ninaE-pect-3xflag flies were labeled with antibodies against FLAG (green) and Calnexin (red). Scale bar is 10 μm. (C-D) Mass-spectrum analysis of PECT enzyme activity. (C) There is no characteristic peak of CDP-Etn in the reaction mixed with a negative control MBP. (D) The product CDP-Etn was detected by the mass spectrum after incubated with PECT recombinant protein. (TIF) [file pgen.1009070.s002.tif]

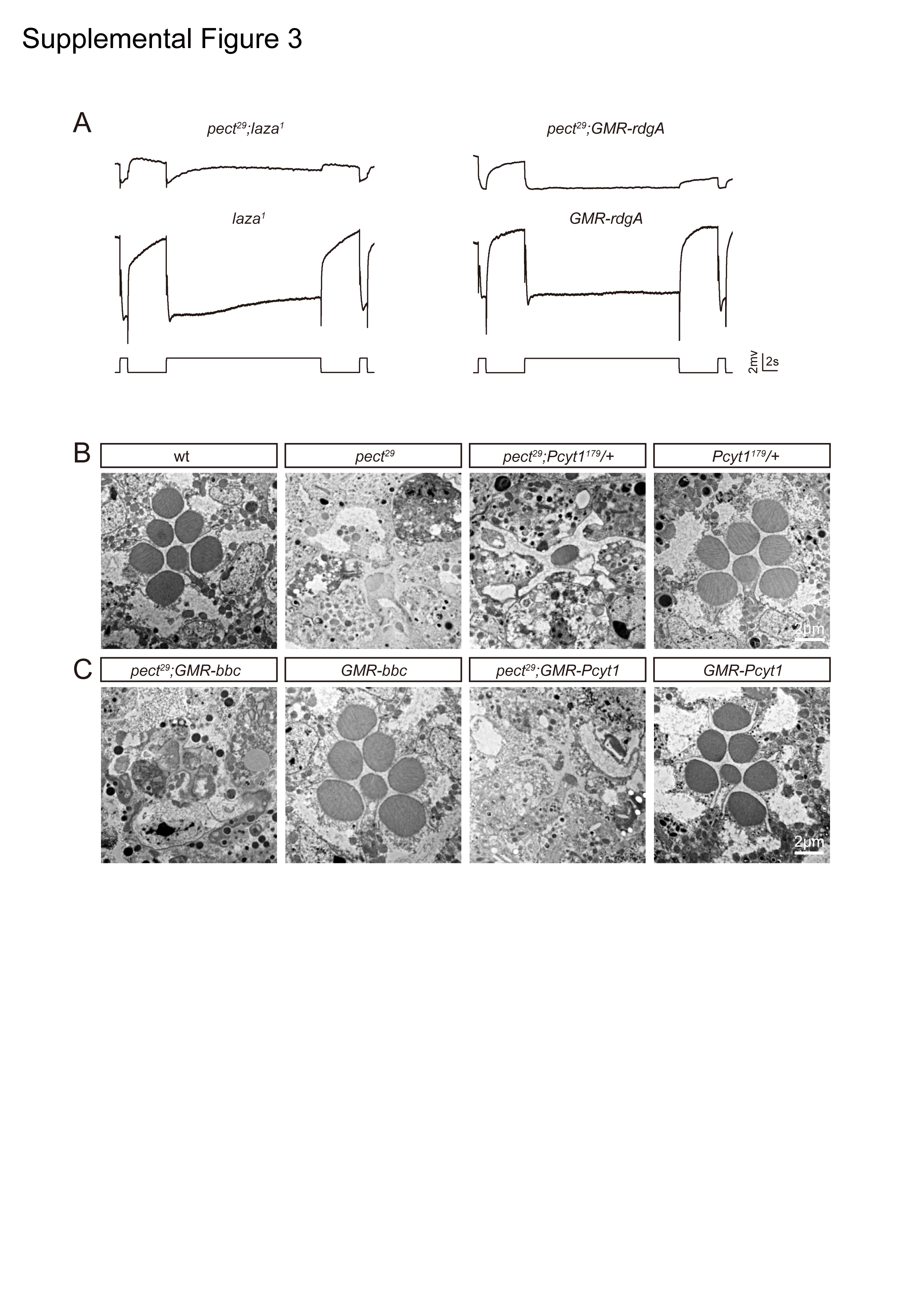

Supplement: S3 Fig — (A) ERG recordings from 1-day-old pect29;laza1, laza1, pect29;GMR-rdgA, GMR-rdgA flies. Flies were dark-adapted for 2 min and subsequently exposed to a 1-s pulse followed by a 20-s then a 1-s pulse of orange light. (B) Reducing PC level did not suppress retinal degeneration in pect29 flies. TEM sections were obtained from wt, pect29, pect29;Pcyt1179/+ (ey-flp rh1-GFP;pect29 FRT40A/GMR-hid CL FRT40A;Pcyt1179/+), and Pcyt1179/+ flies. The Pcyt1 mRNA expression level was reduced to 53% in Pcyt1179/+ flies. Scale bar is 2 μm. (C) Overexpressing PC synthesis enzymes did not further enhance retinal degeneration. TEM sections were obtained from pect29;GMR-bbc (ey-flp rh1-GFP;pect29 FRT40A/GMR-hid CL FRT40A;GMR-bbc/+), GMR-bbc, pect29;GMR-Pcyt1 (ey-flp rh1-GFP;pect29 FRT40A/GMR-hid CL FRT40A;GMR-Pcyt1/+), and GMR-Pcyt1 flies. Scale bar is 2 μm. (TIF) [file pgen.1009070.s003.tif]

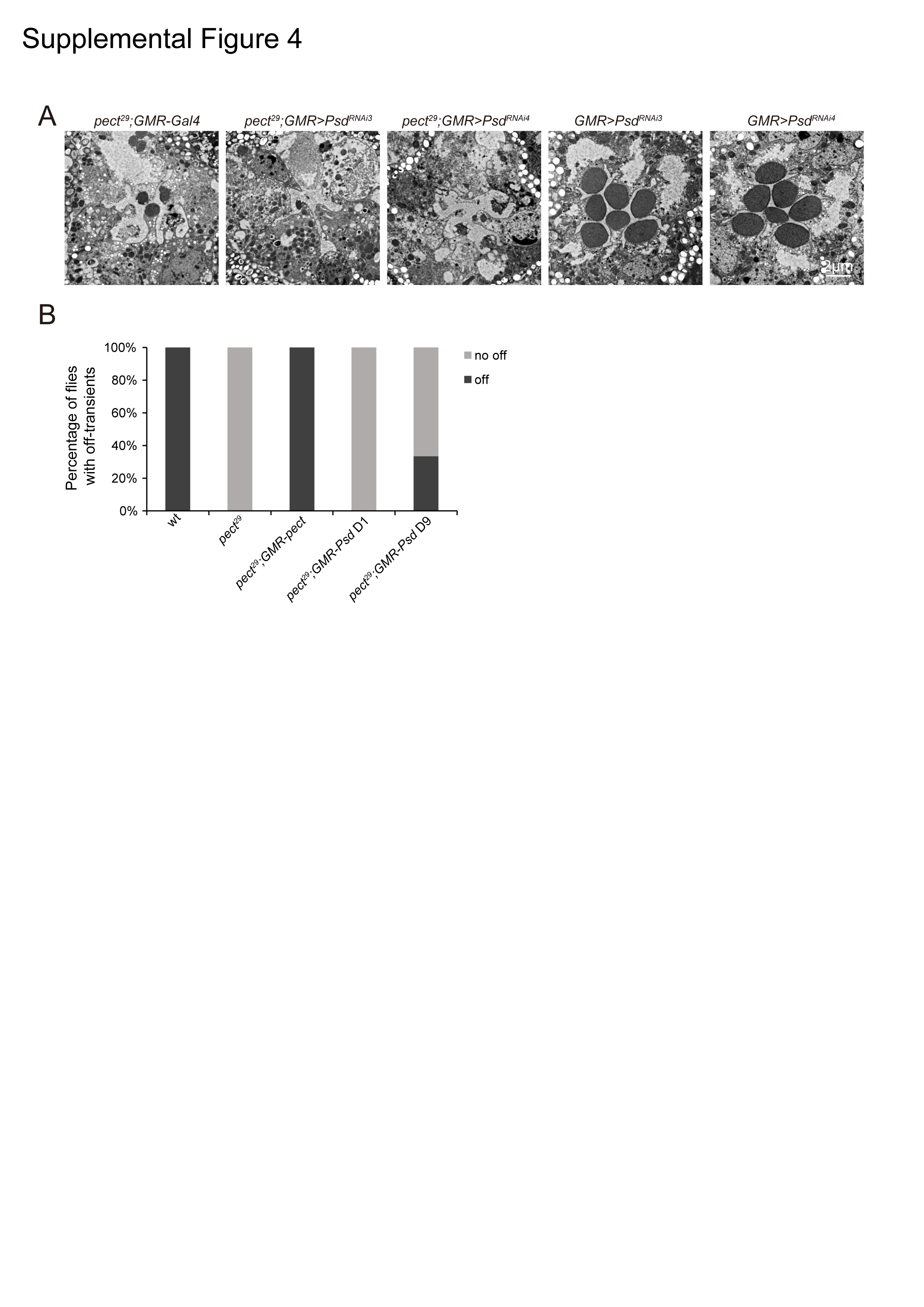

Supplement: S4 Fig — (A) The knockdown of psd enhanced the degeneration phenotype in pect29 mutants. Sections were obtained from pect29;GMR-Gal4, pect29;GMR>PsdRNAi3 (ey-flp rh1-GFP;pect29 FRT40A/GMR-hid CL FRT40A;longGMR-Gal4/UAS-PsdRNAi3), pect29;GMR>PsdRNAi4 (ey-flp rh1-GFP;pect29 FRT40A/GMR-hid CL FRT40A;longGMR-Gal4/UAS-PsdRNAi4), GMR>PsdRNAi3 (longGMR-Gal4/UAS-PsdRNAi3) and GMR>PsdRNAi4 (longGMR-Gal4/UAS-PsdRNAi4). All flies were raised for 5 days under 12h-light/12h-dark cycles. Scale bar is 2 μm. (B) Quantification of off-transients in different genotypes. One third of pect29;GMR-Psd flies display normal off transients on day 9. (TIF) [file pgen.1009070.s004.tif]

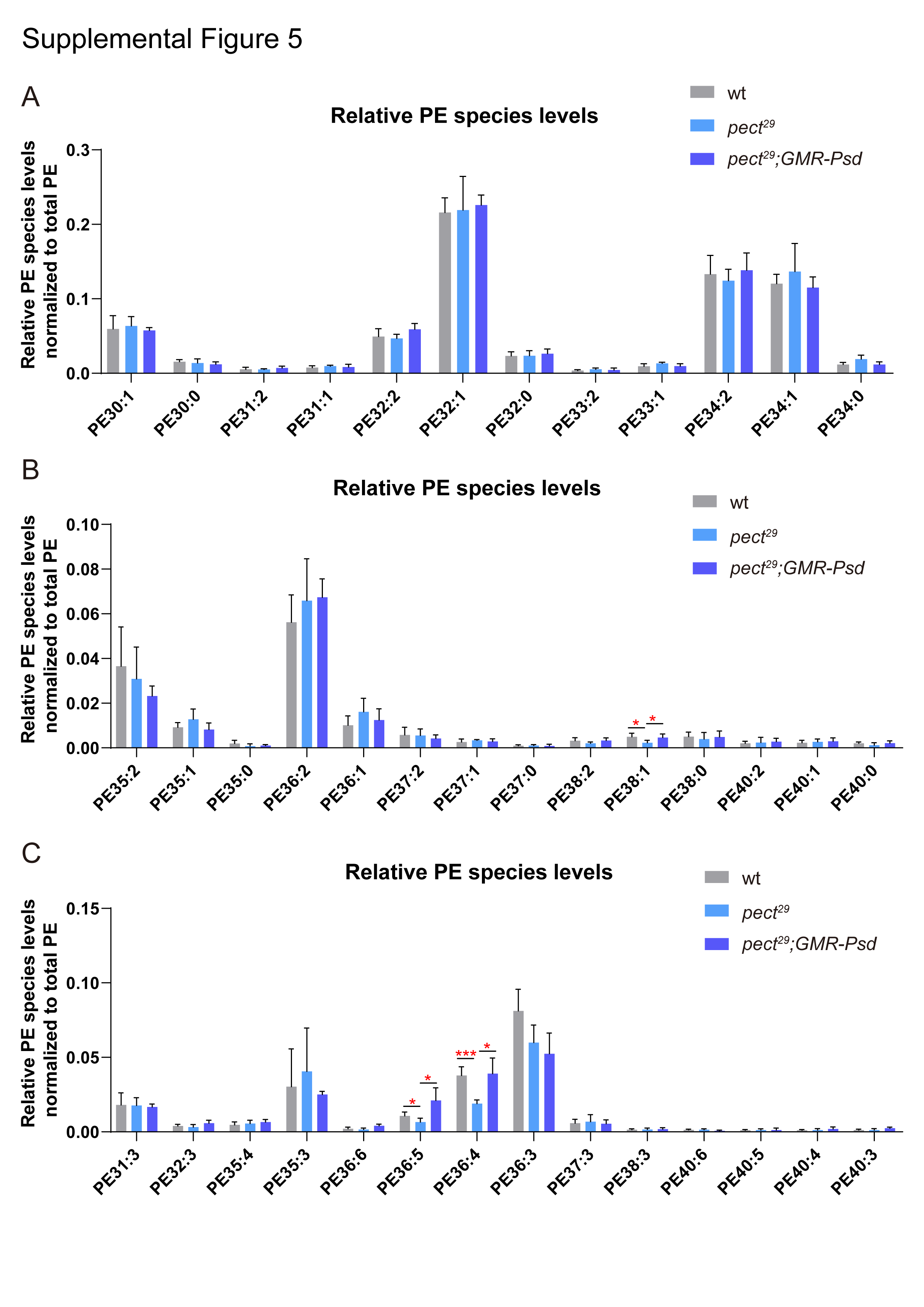

Supplement: S5 Fig — (A-C) Lipidomics analysis of specific PE species levels in genotypes indicated. Specific PE species levels expressed in molar fractions are normalized to total PE levels. Data are presented as mean ± SD, *p < 0.05, ***p < 0.001 (Student’s unpaired t-test). n = 5 replicates of 12 retinas per genotype. (TIF) [file pgen.1009070.s005.tif]

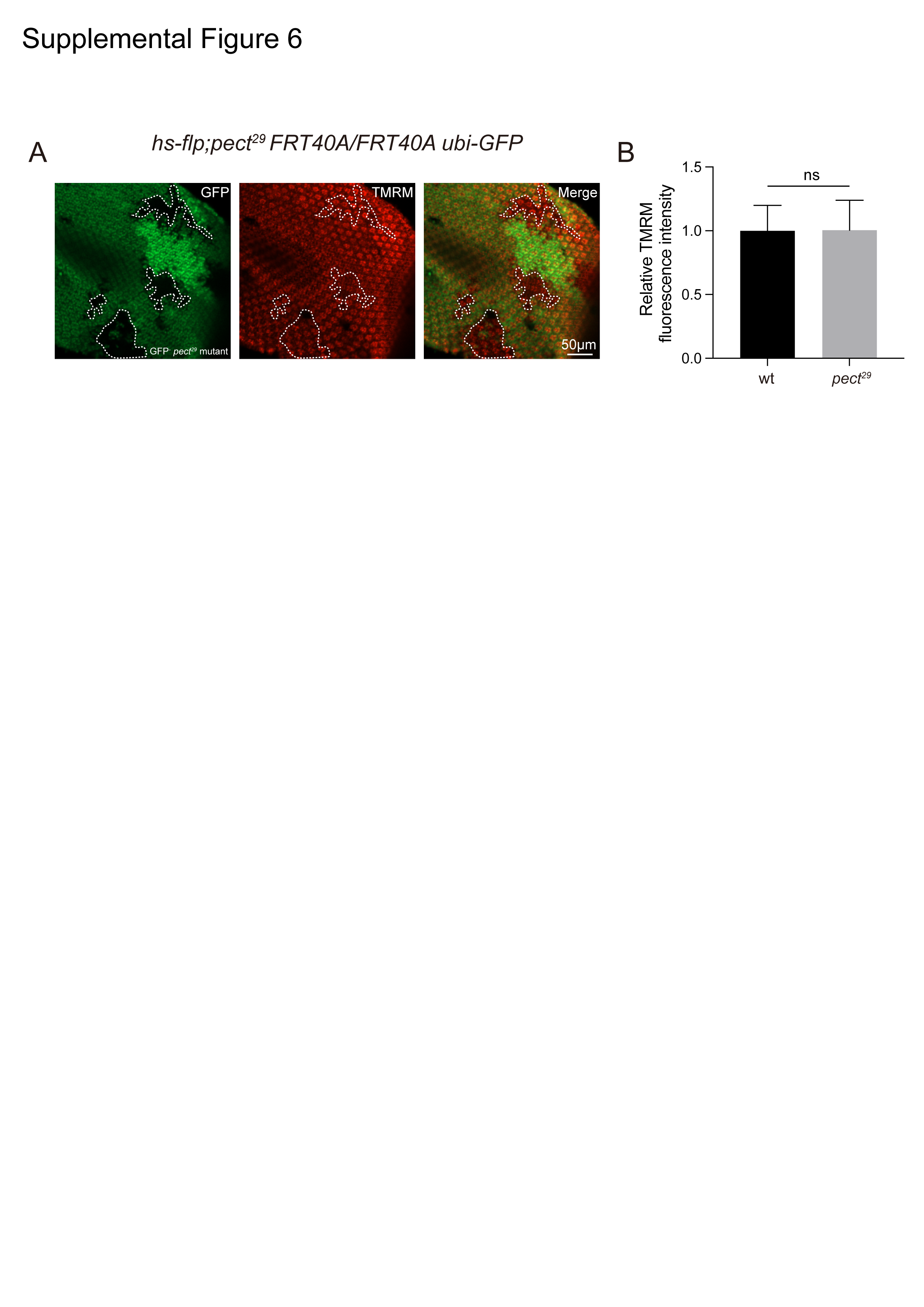

Supplement: S6 Fig — (A) Pupae eyes staining of pect29 mosaic clones from hs-flp;pect29 FRT40A/FRT40A ubi-GFP flies with 100 nM TMRM for 15 min at room temperature, followed by live confocal imaging immediately. GFP negative cells are pect29 mutant cells. Scale bar is 50 μm. (B) Quantification of relative TMRM fluorescence intensity in wild-type and pect29 mutant cells. Data are presented as mean ± SD. ns, not significant (Student’s unpaired t-test). Four different pupae eyes were used for quantification. (TIF) [file pgen.1009070.s006.tif]

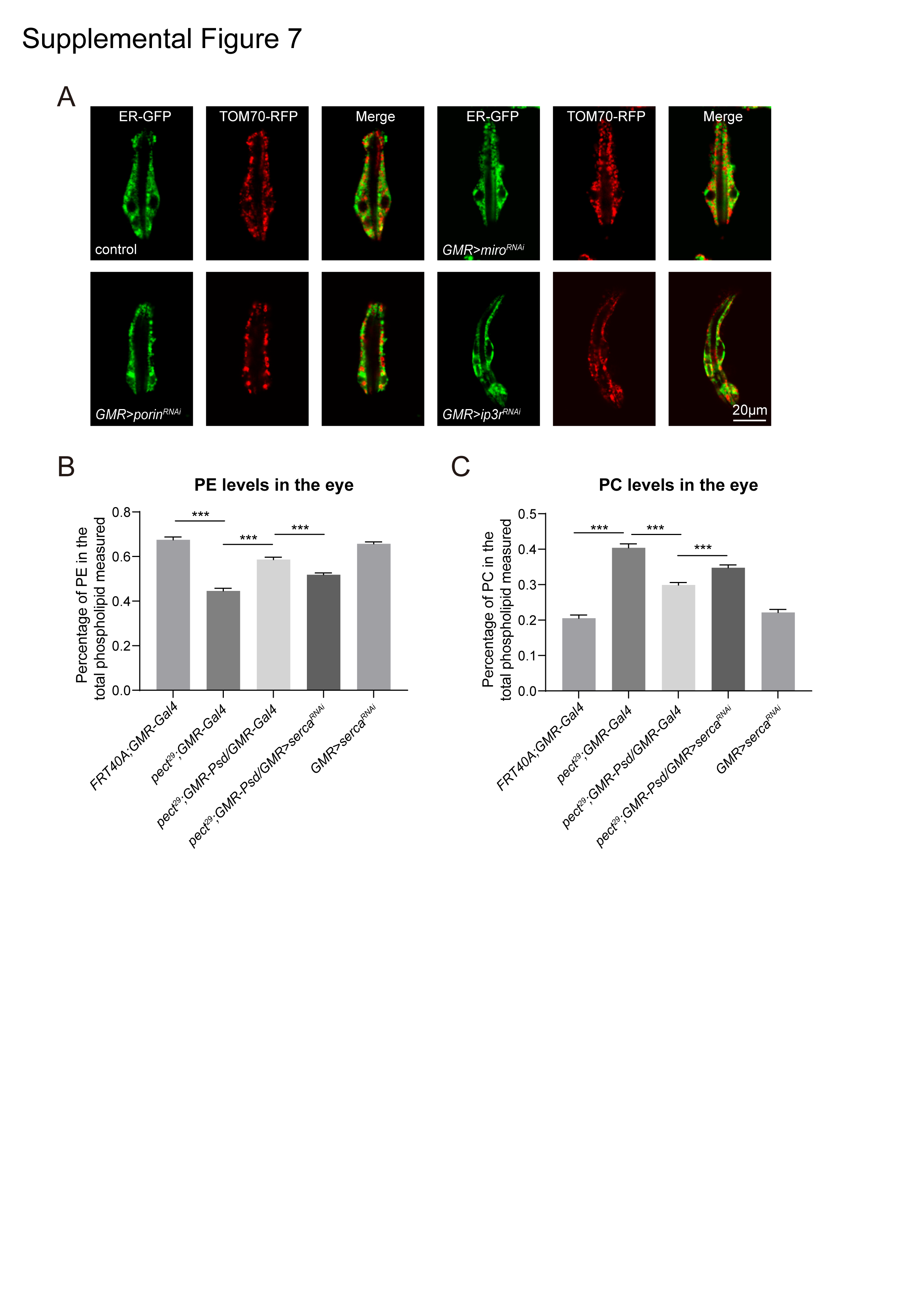

Supplement: S7 Fig — (A) ER-mitochondria contacts are not affected by miroRNAi, porinRNAi, and ip3rRNAi. Live confocal imaging of dissected ommatidia from control (ninaE-KDEL-GFP/+;trp-Tom70-RFP/+), GMR>miroRNAi (longGMR-Gal4/ninaE-KDEL-GFP;UAS-miroRNAi/trp-Tom70-RFP), GMR>porinRNAi (longGMR-Gal4/ninaE-KDEL-GFP;UAS-porinRNAi/trp-Tom70-RFP) and GMR>ip3rRNAi (longGMR-Gal4/ninaE-KDEL-GFP;UAS-ip3rRNAi/trp-Tom70-RFP). Scale bar is 20 μm. (B-C) Lipidomic analysis of retinal PE (B) and PC (C) levels in genotypes indicated. PE and PC levels in molar fractions are normalized to total phospholipids. Data are presented as mean ± SD from 5 replicates of 20 retinas per genotype, ***p < 0.001 (Student’s unpaired t-test). (TIF) [file pgen.1009070.s007.tif]

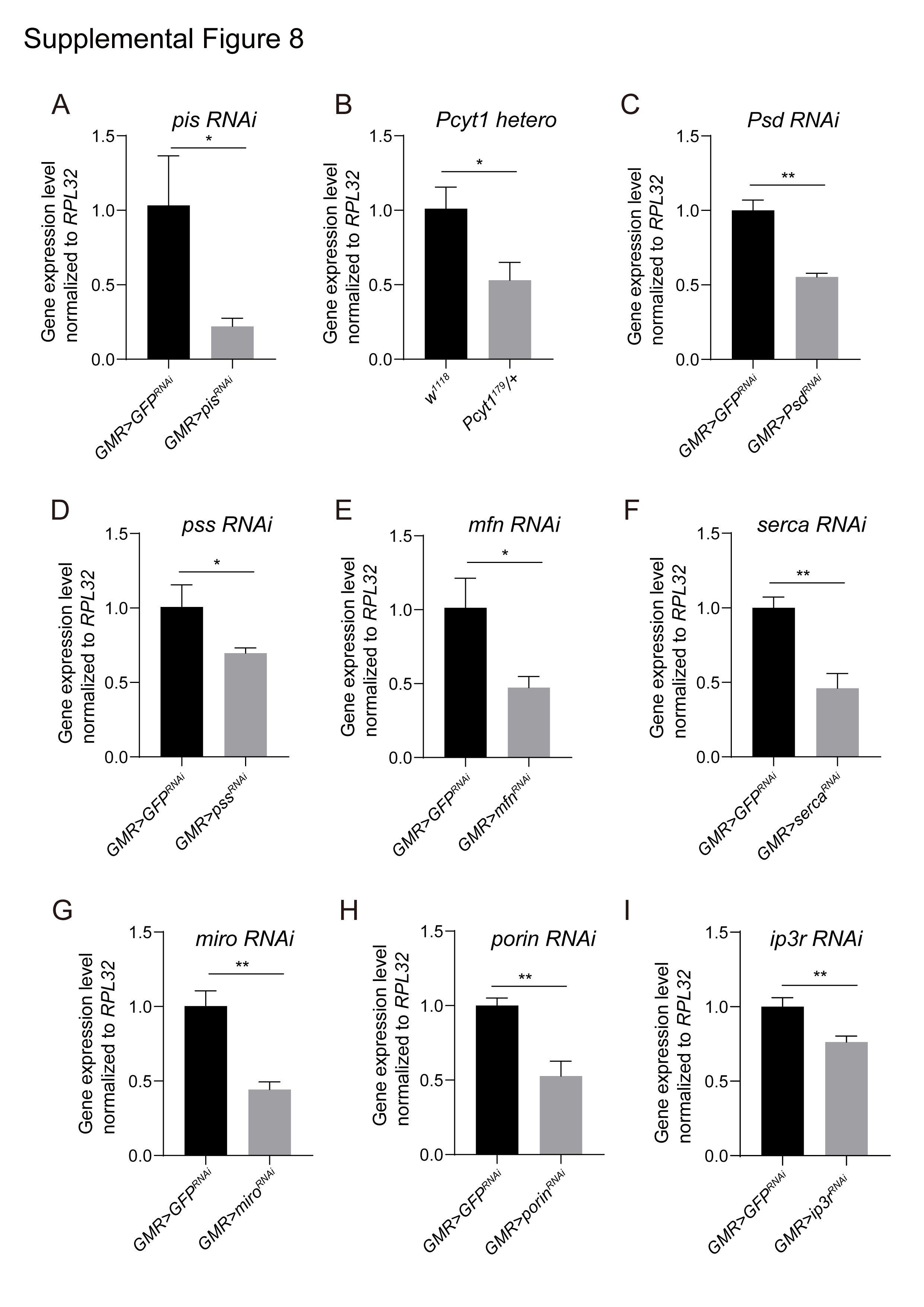

Supplement: S8 Fig — (A-I) RNAi efficiency was determined by using quantitative Real Time PCR (qPCR). Total RNA was extracted from the dissected fly retina of indicated genotypes. The relative expression of target genes was normalized to RPL32, which serves as an internal control. Data are presented as mean ± SD, *p < 0.05, **p < 0.01 (Student’s unpaired t-test). n = 3. (TIF) [file pgen.1009070.s008.tif]
